# Natural transformation drives large-scale genome mosaicism in human gut bifidobacteria

**DOI:** 10.1093/ismejo/wrag208

**Published:** 2026-08-08

**Authors:** Yohei Watanabe, Kento Orihara, Naoki Tsukuda, Taeko Hara, Takahiro Matsuki

**Affiliations:** Food Research Department, Yakult Central Institute, Kunitachi, Tokyo 186-8650, Japan; Basic Research Department, Yakult Central Institute, Kunitachi, Tokyo 186-8650, Japan; Basic Research Department, Yakult Central Institute, Kunitachi, Tokyo 186-8650, Japan; Basic Research Department, Yakult Central Institute, Kunitachi, Tokyo 186-8650, Japan; Basic Research Department, Yakult Central Institute, Kunitachi, Tokyo 186-8650, Japan; Yakult Honsha European Research Center for Microbiology VOF, Gent-Zwijnaarde 9052, Belgium

**Keywords:** human gut microbiome, bifidobacteria, horizontal gene transfer, natural transformation, genome mosaicism, adaptive evolution, genetic engineering, markerless genome manipulation, antibiotic resistance

## Abstract

Although horizontal gene transfer drives bacterial diversification, its contribution to chromosome-scale variation in human gut commensals remains unclear. This study demonstrated that human-associated bifidobacteria undergo extensive chromosomal transfer through natural transformation. Comparative genomics of coexisting *Bifidobacterium pseudocatenulatum* isolates from a single individual revealed extensive recombination signatures between the lineages. We experimentally reproduced this recombination by co-culturing strains, resulting in the transfer of multiple chromosomal regions and generation of mosaic genomes. Individual recombination tracts reached up to 247 kb per site, with cumulative replacements accounting for up to 28.9% of the recipient chromosome. These transfers occurred with heat-killed donors or purified DNA and were abolished by DNase, thereby identifying natural transformation as the underlying mechanism. Furthermore, we observed that environmental factors strongly influenced transformation frequency, suggesting that gut environmental conditions play a role regulating this process. Using natural transformation, we established a simple markerless genome-editing method that enables efficient gene deletions. Deletions of the Tad pili, ComEA–ComEC, or DprA–ComM–YraN gene clusters abolished transformation, defining the core machinery. The conservation of these genes across the genus *Bifidobacterium* and experimental demonstration of natural transformation in *B. longum* and *B. breve* indicate that natural transformation capacity is widespread within the genus. Our findings establish natural transformation as a key mechanism that promotes genome plasticity and contributes to adaptive evolution in bifidobacteria, thereby expanding our current understanding of horizontal gene transfer in the human gut microbiota.

## Graphical Abstract

**Graphical Abstract:**
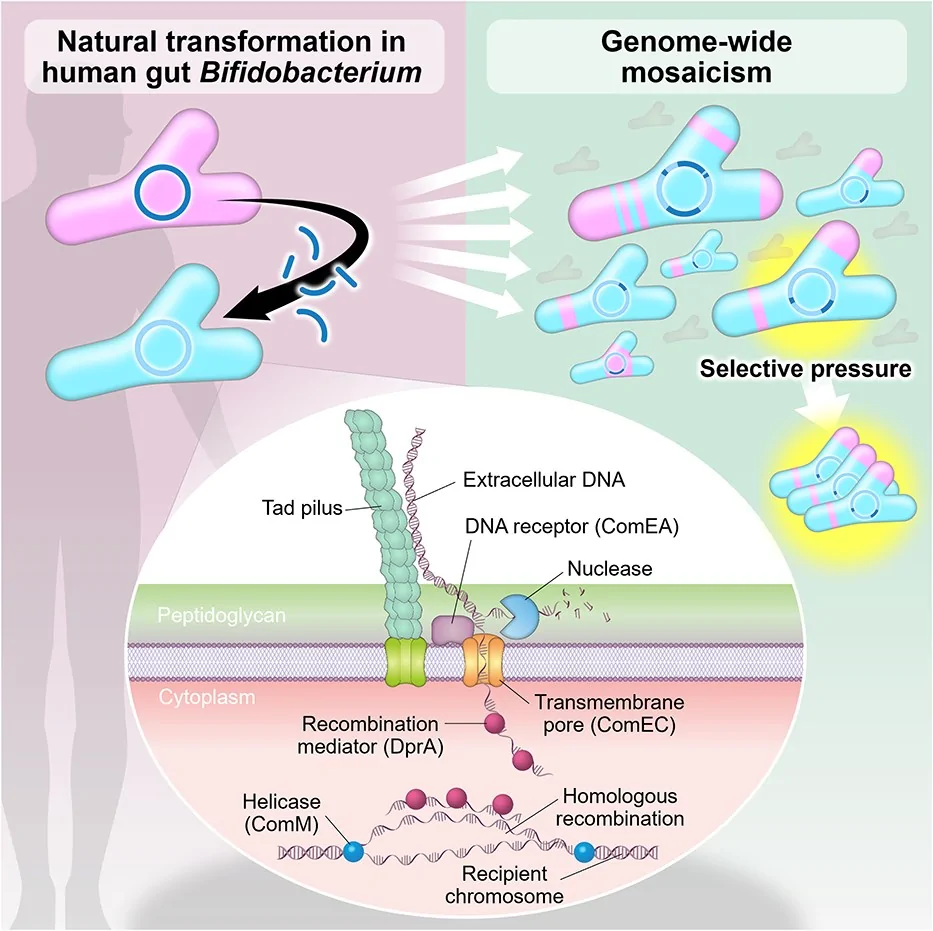

## Introduction

Genomic heterogeneity contributes to phenotypic diversification among bacterial strains within the same species [1]. In the human gut microbiota, strain-level diversity could influence host health by influencing traits such as nutrient metabolism and pathogenicity [2–5]. Genomic heterogeneity arises from both *de novo* mutations and horizontal gene transfer (HGT) [1], the latter of which occurs through mechanisms such as conjugation, transduction, and natural transformation [6]. Although HGT mediated by mobile genetic elements (MGEs), such as plasmids and phages, is well-documented [7], natural transformation, whereby bacteria acquire extracellular DNA for chromosomal integration, remains underexplored in the human gut microbiota.

Bifidobacteria are prominent human gut microbiota members that are associated with host health [8] and exhibit strain-level genotypic and phenotypic heterogeneity [9–11]. Homologous recombination has been estimated to occur more frequently than mutation in bifidobacteria [12], and >10% of genes in many bifidobacterial species are estimated to have been acquired via HGT [13]. Although MGE-mediated HGT has been reported in bifidobacteria [14, 15], such events primarily involve the movement of MGEs themselves and do not readily account for the large-scale chromosomal gene transfer suggested by genomic analyses. Thus, the mechanisms, timescale, and ecological drivers of chromosomal gene transfer in bifidobacteria remain poorly understood.

*Bifidobacterium pseudocatenulatum* is a prevalent human gut commensal [16]. In our previous study, the strain-level distribution of a chromosomal gene encoding a carbohydrate-active enzyme (CAZyme) was incongruent with the species phylogeny, suggesting frequent HGT [9]. We therefore hypothesized that an uncharacterized mechanism facilitates chromosomal gene transfer in *B. pseudocatenulatum*.

In this study, we integrated comparative genomics, co-culture experiments, and genetic manipulation to elucidate the mechanism underlying chromosomal transfer in *B. pseudocatenulatum* and other human-associated bifidobacterial species, and to explore the ecological and evolutionary implications.

## Materials and methods

### Study cohort and ethics

Stool samples were collected from 12 healthy, full-term infants over the first 2 years of their lives, as described previously [17]. The infants were designated A to L. Samples were frozen at −20°C at the home of the infants, transported to the laboratory, and stored at −80°C until analysis. The study followed the Declaration of Helsinki and was approved by the ethics committee of Yakult Central Institute. Written informed consent was obtained from the parents before enrolment.

### *B. pseudocatenulatum* strain isolation

Amplicon sequence variants (ASVs) assigned to *B. pseudocatenulatum* were identified from 1048 previously published 16S rRNA gene amplicon profiles [17] using QIIME2 v2018.11 [18]. ASVs with >97% identity to *B. pseudocatenulatum* RefSeq sequences were selected. From infants harbouring multiple ASVs with distinct temporal dynamics, faecal suspensions were plated on transgalactosylated oligosaccharide (TOS) propionate agar (Yakult Pharmaceutical Industry, Tokyo, Japan) supplemented with 50 μg/ml mupirocin and modified GAM agar (mGAM: Nissui Pharmaceutical, Tokyo, Japan) supplemented with 0.5% (w/v) lactose and 50 μg/ml mupirocin. Plates were incubated at 37°C for 2 days in an anaerobic chamber (Coy Laboratories, MI, USA) with 88% N_2_, 7% H_2_, and 5% CO_2_. Single colonies were isolated, and *B. pseudocatenulatum* was identified by colony polymerase chain reaction (PCR) using species-specific primers [16]. Isolates were further classified by random amplified polymorphic DNA profiling [19]. For genome sequencing, one to three strains per lineage were selected.

### Culture conditions

Unless otherwise stated, strains were grown anaerobically in 0.5% (w/v) lactose-supplemented mGAM broth (mGAML) at 37°C for 16 h.

### Genome sequencing, assembly, and annotation

For short-read sequencing, genomic DNA was extracted using a glass-bead phenol method [9]. Genomic libraries were prepared using the TruSeq DNA PCR-free Library Preparation Kit (Illumina) and sequenced via the MiSeq platform, using the v2 reagent kit (500 cycles; Illumina).

For long-read sequencing, high-molecular-weight DNA was extracted using a NucleoBond AXG20 with buffer set III (Macherey-Nagel, Düren, Germany). Libraries were prepared using Oxford Nanopore ligation sequencing kits and sequenced on a MinION device with an FLO-MIN106 flow cell (Oxford Nanopore Technologies).

Hybrid genome assembly was performed using Unicycler v0.4.8 [20] with default settings and genome annotation was conducted using Prokka v1.13 [21] with default parameters. Prophage regions were identified using PHASTER [22]. Integrative and conjugative elements (ICEs) and integrative mobilizable elements (IMEs) were identified using ICEfinder [23].

### Core-gene recombination candidate identification

Core genes of strains from the same host were identified using Roary v3.13 [24] with default settings. The core-genome alignment produced by Roary was used to construct a phylogenetic network with SplitsTree [25], enabling the identification of isolate pairs with evidence of core-gene recombination.

### Recombination region identification and donor-concordant SNP extraction

Donor and recombinant genomes were aligned using Snippy v4.6.0 (https://github.com/tseemann/snippy) with the recipient genome as the reference. Recent recombination tracts were inferred using fastGEAR [26]. To provide complementary support for inferred imports, variants in the donor and transformant were called against the recipient genome, and donor-concordant single-nucleotide polymorphisms (SNPs) were defined as positions at which the donor and recombinant shared the same allele that differed in the recipient. Recombination tracts and donor-concordant SNPs were visualized using Circos v0.69-8 [27]. Gene-level synteny within representative recombination tracts was visualized using pyGenomeViz (https://github.com/moshi4/pyGenomeViz). Pairwise SNP density between donor and recipient genomes was calculated from the Snippy output. Biallelic SNPs were counted from the VCF file after excluding indels, complex variants, and multiallelic sites. The denominator was defined as the number of unambiguous A/C/G/T positions in the Snippy aligned consensus sequence, and SNP density was expressed as SNPs per kilobase.

### Chromosomal transfer assay

Donor and recipient strains were grown to late exponential phase in mGAML broth, washed, and resuspended in modified peptone-yeast extract (mPY) medium [9]. Equal volumes of donor and recipient suspensions were mixed; thereafter, 10 μl samples were spotted onto mGAM agar and incubated anaerobically for 16 h. Cells were transferred to appropriate selective conditions, and recombinant colonies were isolated and analysed via whole-genome sequencing. The donor–recipient strain combinations used in the chromosomal transfer assays are provided in the supplementary information (Table S1). Three chromosomal transfer assays were performed as follows:

(i) ***BpXyn10A* transfer**  *BpXyn10A* is a chromosomally encoded glycoside hydrolase family 10 (GH10) endo-1,4-β-xylanase gene [9]. Donor strains carried *BpXyn10A* and were susceptible to tetracycline, whereas recipient strains lacked *BpXyn10A* and were resistant to tetracycline. Recombinants were selected on mPY medium supplemented with 0.5% (w/v) arabinoxylan and 5 μg/ml tetracycline.
(ii) **Starch utilization transfer** Donor strains exhibited starch utilization and were susceptible to tetracycline, whereas recipient strains lacked starch utilization and were resistant to tetracycline. Selection was performed on mPY medium supplemented with 0.5% (w/v) soluble starch and 5 μg/ml tetracycline.
(iii) ***tetW* transfer** Donor strains carried *tetW* and were susceptible to erythromycin, whereas recipient strains lacked *tetW* and were resistant to erythromycin. Recombinants were selected on mGAML agar containing 5 μg/ml tetracycline and 1 μg/ml erythromycin. Recipient colony-forming units (CFUs) were enumerated on mGAML agar containing 1 μg/ml erythromycin. The recombinant recovery frequency was calculated as the number of tetracycline- and erythromycin-resistant recombinant CFUs divided by the number of erythromycin-resistant recipient CFUs.

### Chromosomal transfer mode identification

To distinguish natural transformation from conjugation or transduction, tetracycline-resistance transfer assays were repeated with (i) live donors, (ii) heat-killed donors (80°C for 10 min), or (iii) high-molecular-weight donor genomic DNA. Where indicated, DNase I (Takara Bio, Otsu, Japan) was added to the donor (YIT 4072^T^)-recipient (YIT 12824) mixtures at a final concentration of 1 U per 10 μl. Recombinant colonies were isolated, and selected isolates were subjected to whole-genome sequencing. As an additional phenotype-based assay, purified donor genomic DNA was also used to assess the transfer of starch-utilization ability. Recipient (YIT 12819) cells were mixed with high-molecular-weight genomic DNA from a starch-utilizing donor strain (YIT 11027), incubated on mGAM agar, and subsequently selected on mPY medium supplemented with 0.5% (w/v) soluble starch.

### Universality of natural transformation in *B. pseudocatenulatum*

We PCR-amplified an ~20 kb *tetW*-carrying chromosomal fragment from YIT 4072^T^ genomic DNA using primers BpTy20k-F (TCCTGTGGATTTCGACGGTGACGAGCAG) and BpTy20k-R (GAGTGCGGTTGATGGTGCATACGGCAAG), together with PrimeSTAR GXL DNA polymerase (Takara Bio, Otsu, Japan). The DNA fragment was ethanol-precipitated, resuspended in TE buffer at a final concentration of 1 ng/μl, and mixed with the recipient cells (Table S2) as described above. After incubation on mGAM agar, transformants were selected on mGAML agar containing 5 μg/ml tetracycline.

### Long-arm donor DNA preparation

Donor DNA with extended homologous arms was generated via a two-step strategy. First, a short-arm linear construct containing YIT 4072^T^-derived *tetW* was generated using overlap-extension PCR (OE-PCR) and introduced into an intergenic region of the YIT 12819 chromosome via natural transformation on mGAM agar supplemented with 50 mM MgCl_2_, yielding a tetracycline-marked derivative (YIT 12819Tc). Second, the YIT 12819Tc genome was used as a template to amplify a long-arm linear donor fragment. This fragment (consisting of an 8.3 kb upstream homologous arm, 2.6 kb *tetW*, and a 9.1 kb downstream homologous arm) was amplified using PrimeSTAR GXL DNA polymerase. The resulting long-arm donor DNA (final concentration: 1 ng/μl in TE buffer) was used for transformation frequency assays. Further details are provided in the supplementary methods, and the primers used for long-arm donor DNA preparation are listed in Table S5.

### Transformation frequency quantification

Recipient cell suspensions were mixed with the long-arm donor DNA, and 10 μl aliquots were spotted onto mGAM agar. After 16 h anaerobic incubation at 37°C, cells were recovered and plated onto tetracycline-containing selective and nonselective mGAML agar for CFU enumeration. Transformation frequency was calculated as the ratio of CFUs on selective plates to those on nonselective plates.

To assess the effects of environmental factors on transformation frequency, mGAM agar was used as the base medium and supplemented with individual additives, including fermentable sugars (e.g. lactose, maltose, and sucrose), organic acids (acetate:lactate:formate at a ratio of 8:3:2), sodium salts of these acids, inorganic salts (NaCl, KCl, NH_4_Cl, and MgCl_2_), pH-modulating agents (HCl or NaOH), or non-fermentable sugar fucose. Effects were summarized as changes in transformation frequency relative to the control condition.

Unless otherwise indicated, five biological replicates were performed per condition. Statistical analyses were conducted using R version 4.3.2 (31 October 2023). Transformation frequencies were compared using two-sided Mann–Whitney *U* tests, with *P* < .05 considered statistically significant. Values below the limit of detection were assigned a value equal to one-half the detection limit for statistical analyses. Where applicable, data are presented as individual values with the mean ± standard deviation.

### RNA sequencing analysis

YIT 12819 cells were spotted onto (i) control mGAM agar, (ii) transformation-suppressing medium (mGAM agar adjusted to pH 6.0), or (iii) transformation-promoting medium (mGAM agar supplemented with 50 mM MgCl_2_), and incubated anaerobically at 37°C for 16 h. Total RNA was extracted using the hot-phenol method, treated with DNase I, and further purified via phenol–chloroform–isoamyl alcohol extraction and ethanol precipitation. RNA integrity was assessed on a Bioanalyzer 2100 (Agilent, Santa Clara, CA, USA). All samples exhibited RNA integrity numbers (RINs) greater than nine. Ribosomal RNA was depleted using QIAseq FastSelect-5S/16S/23S. Strand-specific libraries were prepared using the QIAseq Stranded RNA Library Kit (Qiagen, Hilden, Germany). Sequencing was performed on an Illumina MiSeq System using the MiSeq Reagent Kit v3 (150 cycles), generating 75 bp paired-end reads.

Raw reads were quality-filtered using fastp v0.23.2 [28] with parameters of (-q 30 -l 51 --cut_right_mean_quality 30 -n 0 -e 30 -w 8). Residual rRNA was removed using SortMeRNA v4.3.4 [29] with default parameters and mapped to the YIT 12819 reference genome using Bowtie2 v2.4.0 [30] with default settings. Read counts per gene were obtained using featureCounts v2.0.1 [31] with default settings. Genes and gene clusters differentially expressed between transformation-promoting and transformation-suppressing conditions were identified (Table S3). Genes with ≥10-fold increase in transcript abundance under the MgCl_2_-supplemented condition compared with the pH 6.0 condition were further analysed.

### Markerless genome manipulation

*upp*-encoded uracil-phosphoribosyltransferase (UPRTase) converts 5-fluorouracil (5FU) into toxic metabolites. Therefore, *upp* functions as a negative selection marker in the presence of 5FU [32]. A markerless genome manipulation method relying on the *upp*/5FU counterselection system and natural transformation, originally developed in *Bacillus subtilis* [32], was adapted to *B. pseudocatenulatum*. The overall workflow is outlined below; detailed protocols are provided in the supplementary methods, and the primes used are listed in Tables S6–S8.

### *upp* deletion mutant construction

Because *upp* is conserved among bifidobacteria, the YIT 12819 *upp* deletion mutant was generated. Approximately 3.4 kb upstream and 3.5 kb downstream regions flanking *upp* were amplified and OE-PCR-joined to create a linear deletion construct. This fragment was introduced into YIT 12819 via natural transformation under transformation-promoting conditions, and transformants were selected on mGAML agar containing 5FU (100 μg/ml). Colonies resistant to 5FU were screened using colony PCR and Sanger sequencing. A confirmed *upp* deletion strain was designated YIT 12819Δ*upp*.

### *upp*–*tetW* counterselection cassette preparation

For positive selection and subsequent marker excision, a counterselection cassette was constructed by inserting the YIT 4072^T^ strain-derived *tetW* gene into an intergenic region downstream of *upp* in the YIT 12819 genome. The *tetW-*containing construct was introduced into YIT 12819 via natural transformation, and tetracycline-resistant colonies were selected. Genomic DNA from a confirmed strain was used as a template to amplify a 3.6 kb linear *upp*–*tetW* cassette for subsequent genome manipulation.

### Markerless deletion of target genes

For markerless gene deletion, 1–2 kb upstream and downstream homologous arms of the target gene were amplified. These fragments and the *upp–tetW* cassette were OE-PCR-joined, with the downstream arm containing a short direct repeat sequence shared with the upstream arm. The resulting linear construct was introduced into YIT 12819Δ*upp* via natural transformation, and the insertion mutants were selected on tetracycline-containing medium. To excise the *upp*–*tetW* cassette, insertion strains were subsequently cultured without selection and plated onto 5FU-containing agar, enabling counterselection for recombination between the direct repeats. Colonies having lost both the target gene and the *upp*–*tetW* cassette were identified via colony PCR and verified by Sanger sequencing. Strains exhibiting the expected genotype were used as markerless deletion mutants.

### Deletion mutant transformation frequency

Transformation efficiency of the deletion mutants was measured by incubating bacterial cells with the long-arm donor DNA on mGAM agar supplemented with 50 mM MgCl_2_. Experiments were performed in five biological replicates. The differences were analysed using the Mann–Whitney *U* test.

### Natural transformation gene conservation in *Bifidobacterium* species

Representative complete genomes of *Bifidobacterium* species were downloaded from the NCBI RefSeq. Homologs of genes essential for natural transformation were identified using Gcluster [33] with default parameters. A phylogenetic tree was constructed based on 16S rRNA gene sequences using the Maximum Likelihood method in MEGA X [34]. Although the *yraN–comM–dprA* operon was absent in the representative *Bifidobacterium subtile* (GCF_014898195.1) genome, it was present in another genome of the same species (GCF_000741775.1). *Bifidobacterium subtile* analyses are thus based on GCF_000741775.1.

### Chromosomal transfer assays in *B. longum* and *B. breve*

For *B. longum* subsp. *longum*, donor and recipient strains differing in galactan utilization and erythromycin resistance were mixed at a 1:1 ratio, spotted onto mGAM agar, and incubated anaerobically overnight. Recombinants were selected in an erythromycin-containing mPY medium, with galactan serving as the sole carbon source.

For *B. breve*, heat-killed erythromycin-resistant donor cells or purified donor genomic DNA were mixed with erythromycin-susceptible recipients, spotted onto mGAM agar, and incubated overnight. Recombinant colonies were selected on erythromycin-containing mGAML agar and confirmed by strain-specific PCR. Selected recombinant isolates were then subjected to whole-genome sequencing.

## Results

### Chromosomes of coexisting *B. pseudocatenulatum* isolates contain recombination signatures

To search for genomic signatures of recent chromosomal transfer within individual hosts, we longitudinally isolated *B. pseudocatenulatum* strains from the same infants. Faecal samples were collected from 12 infants during the first 2 years of their lives [17] and ASV analysis identified infants H, J, and K as harbouring multiple *B. pseudocatenulatum* ASVs with distinct temporal dynamics (Fig. 1A and B). Infants B and F also harboured multiple ASVs; however, these exhibited nearly identical temporal profiles, suggesting intragenomic 16S rRNA gene sequence variation rather than distinct coexisting lineages. From infants H, J, and K, we isolated 33 *B. pseudocatenulatum* strains and sequenced their genomes (Fig. 1B).

**Figure 1:**
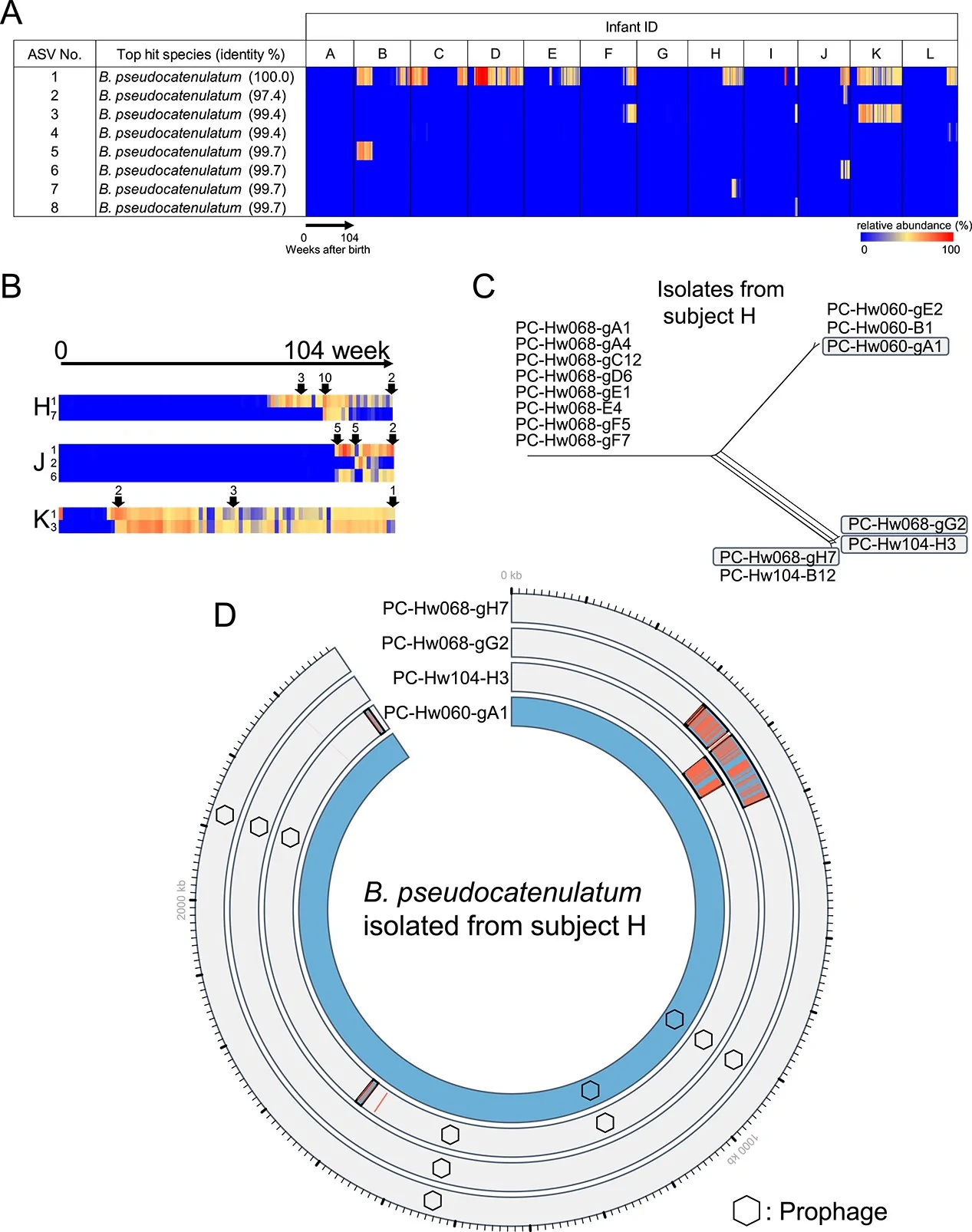
Chromosomal recombination among coexisting *B. pseudocatenulatum* lineages within individual hosts (A) longitudinal dynamics of *B. pseudocatenulatum* ASVs in faecal samples collected from infants during the first 2 years of their lives. The data are represented as a heatmap across 1048 samples from 12 infants (A–L). Columns: samples ordered by sampling time within each infant. Rows: ASVs. Colour intensity: relative abundance. (B) Overview of strain isolation from infants harbouring multiple *B. pseudocatenulatum* ASVs with distinct temporal patterns. Arrows: sampling time points at which *B. pseudocatenulatum* strains were isolated. Numbers above the arrows: number of isolates subjected to whole-genome sequencing at each time point. (C) Phylogenetic network of strains isolated from infant H, reconstructed using core-genome SNPs. (D) Recombination tracts between coexisting lineages are inferred using fastGEAR, detecting recent homologous recombination events between bacterial genomes. Shaded segments in PC-Hw068-gG2 and PC-Hw104-H3: fastGEAR-inferred putative donor-derived chromosomal tracts. Vertical lines: putative donor-concordant SNPs. Hexagons: prophage regions.

In infant H, core-genome SNP-based phylogenetic network analysis identified three lineages and revealed reticulate relationships among strains of different lineages (Fig. 1C), suggesting recombination among lineages. To identify recent recombination events, we applied fastGEAR [26], which can infer recent chromosomal imports, and detected discrete recombination tracts ranging from 4 to 108 kb between coexisting lineages (Fig. 1D). Strains PC-Hw060-gA1 and PC-Hw068-gH7 were found to serve as the putative donor and recipient, respectively (Fig. 1D). Strains PC-Hw068-gG2 and PC-Hw104-H3 were identified as recombinant strains carrying a chromosomal segment inferred to originate from strain PC-Hw060-gA1. Thus, recent recombination tracts were detected in 2 of the 33 sequenced isolates, both from infant H at 68 and 104 weeks. Because isolates from this infant were available from only three time points (60, 68, and 104 weeks), the precise timing of the recombination events and whether they accumulated over time could not be inferred. To visualize these recombination signals more explicitly, we identified putative donor-concordant SNPs, defined as positions at which the putative recombinant shared the donor but not the recipient allele. Dense clusters of these SNPs coincided with the donor tracts inferred by fastGEAR, providing a clear visual representation of recent chromosomal transfer (Fig. 1D). To further evaluate the robustness of these inferred recombination tracts, we visualized both donor-concordant and donor-discordant SNPs within representative fastGEAR-inferred regions. Donor-concordant SNPs were densely distributed throughout the donor-inferred imports, whereas donor-discordant SNPs were rare and scattered (Fig. S1). Although prophage acquisition events were detected in these genomes, the inferred recombination tracts were located distal to prophage integration sites (Fig. 1D), and no evidence of plasmid acquisition was observed. These observations indicate that the major mosaic segments are unlikely to result from plasmid- or phage-mediated transfer but instead reflect recent homologous recombination among coexisting *B. pseudocatenulatum* lineages within the same host.

### *In vitro* chromosomal transfer between the strains

To experimentally reproduce chromosomal transfer under controlled conditions, we co-cultured *B. pseudocatenulatum* strains at high cell density, thereby increasing the likelihood of HGT. We focused on *BpXyn10A*, a chromosomally encoded glycoside hydrolase family 10 (GH10) gene encoding for endo-1,4-β-xylanase, involved in long-chain xylan (LCX) degradation and previously suggested to be horizontally transferred [9]. From our strain collection, we selected isolates that either carried *BpXyn10A* or lacked it but exhibited tetracycline resistance (Fig. 2A and Table S4). We mixed and co-cultured each donor–recipient pair on nonselective agar for 16 h. We subsequently inoculated the resulting cell mixtures into LCX- and tetracycline-containing liquid medium as selective pressure, leading to the emergence of strains exhibiting both LCX utilization and tetracycline resistance (Fig. 2A and Table S1). Whole-genome sequencing of these strains, followed by fastGEAR analysis, identified recombination tracts harbouring *BpXyn10A*, which largely overlapped dense clusters of donor-concordant SNPs (Fig. 2B and C). We detected no plasmid acquisition or new prophage integration. Recombination tracts inferred by fastGEAR coincided with donor-concordant SNP clusters, closely mirroring the recombination signatures observed in the infant-derived isolates. Recombination patterns differed among independent recombinants isolated from the same donor–recipient co-culture, with variable breakpoints and, in certain strains, additional recombination events at *BpXyn10A*-unrelated loci (Figs 2B and S2). These findings demonstrate that chromosomal segments were MGE-independently transferred on agar surfaces, enabling the emergence of *BpXyn10A*-acquiring strains under selective conditions.

**Figure 2:**
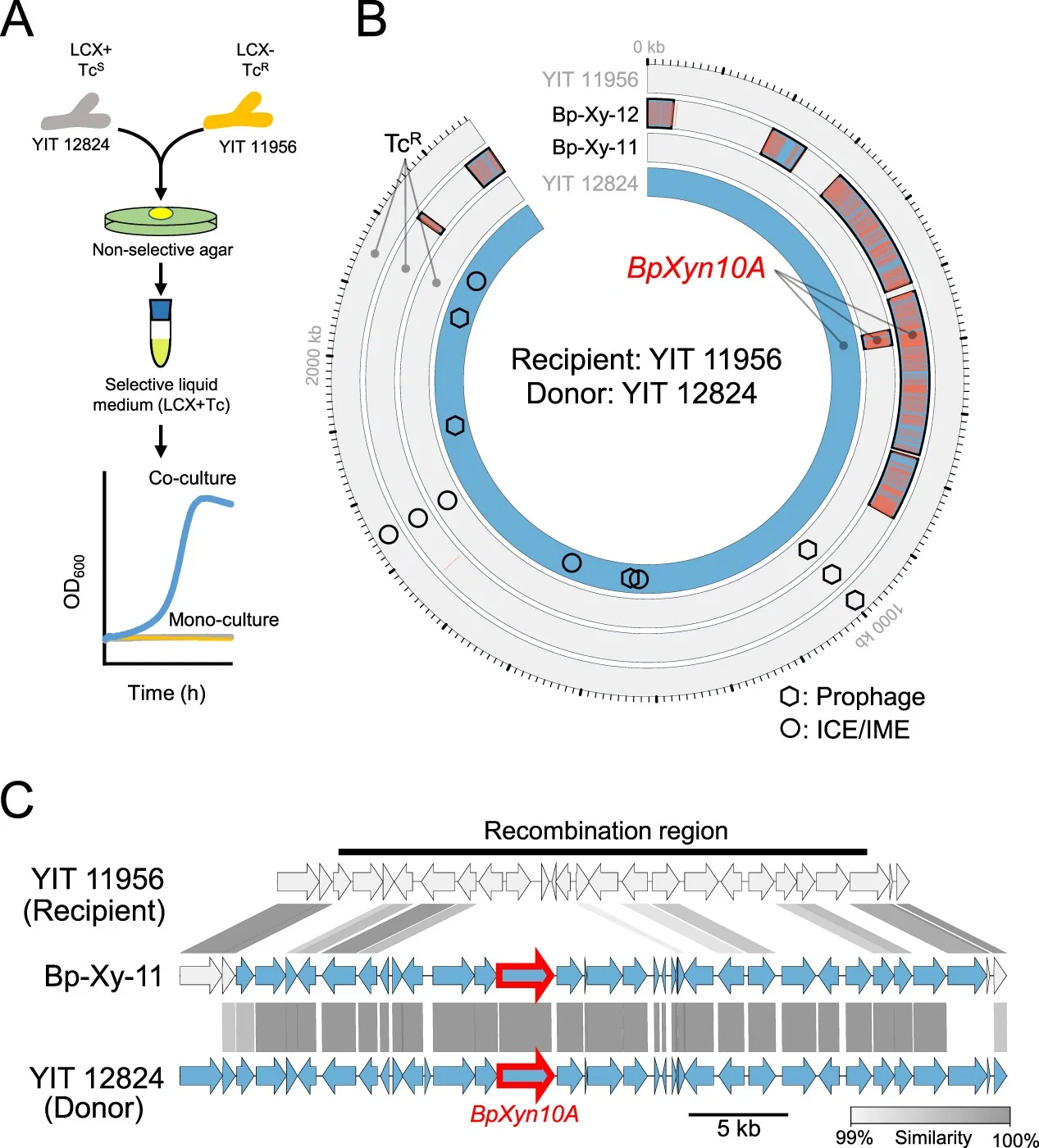
Horizontal transfer of *BpXyn10A* via homologous recombination between co-cultured *B. pseudocatenulatum* strains. (A) Experimental overview of *in vitro* HGT assays. Strains exhibiting different phenotypes were independently cultured in liquid medium, mixed, and co-cultured on nonselective agar for 16 h. The resulting cell populations were inoculated into LCX- and tetracycline-containing selective media. Growth under selective conditions, in which neither parental strain alone could thrive, was interpreted as the emergence of recombinant strains. “LCX+” and “LCX−”: presence or absence of the LCX utilization gene, respectively. “S/R”: antibiotic sensitivity or resistance. Representative results are shown for pairing with YIT 11956 and YIT 12824 as the recipient and donor, respectively. (B) Recombination events in representative recombinant strains (Bp-Xy-11/12) simultaneously obtained from YIT 11956 and YIT 12824 as the recipient and donor, respectively. Shaded segments in Bp-Xy-11 and Bp-Xy-12: fastGEAR-inferred donor-derived chromosomal tracts. Vertical lines: donor-concordant SNPs. Hexagons and circles: phage and ICE/IME insertion sites, respectively. **(**C) A donor-derived chromosomal segment harbouring *BpXyn10A* in the recombinant strain Bp-Xy-11. Blue arrows: fastGEAR-inferred donor-derived genes. Links between gene arrows: amino acid sequence similarity between proteins. LCX, long-chain xylan; Tc, tetracycline; ICE, integrative and conjugative element; IME, integrative and mobilizable element. See also Fig. S2 and Table S1 for all donor–recipient combinations and corresponding recombinants.

### Diverse phenotypic traits are transferred via chromosomal recombination

To determine whether chromosomal recombination mediates the transfer of phenotypic traits in addition to LCX utilization, we applied different selective pressures based on starch utilization and tetracycline resistance, both of which vary among strains in our collection (Table S2). Following co-culture on nonselective agar for 16 h, we obtained recombinant isolates exhibiting the selected phenotypes (Fig. 3A and B; Table S1). In the tetracycline-resistance transfer assays, tetracycline-resistant recombinants were obtained in three independent experiments at frequencies of 2.5 × 10^−6^, 1.2 × 10^−7^, and 7.9 × 10^−7^ per recipient CFU. Whole-genome sequencing of these recombinants identified phenotype-associated chromosomal segments integrated into the recipient genome, consistent with the above-described recombination patterns (Figs 3C, D, S3A, and  B). Changing the selective pressure within the same donor–recipient pair resulted in the selection of recombinants that had acquired distinct chromosomal segments (Fig. 3E). When YIT 11027 and YIT 11956 were co-cultured, LCX-based selection yielded recombinants carrying tracts harbouring the *BpXyn10A* region, whereas starch-based selection enriched for a distinct chromosomal tract harbouring a gene that encodes an α-amylase-family glycoside hydrolase of GH13.

**Figure 3:**
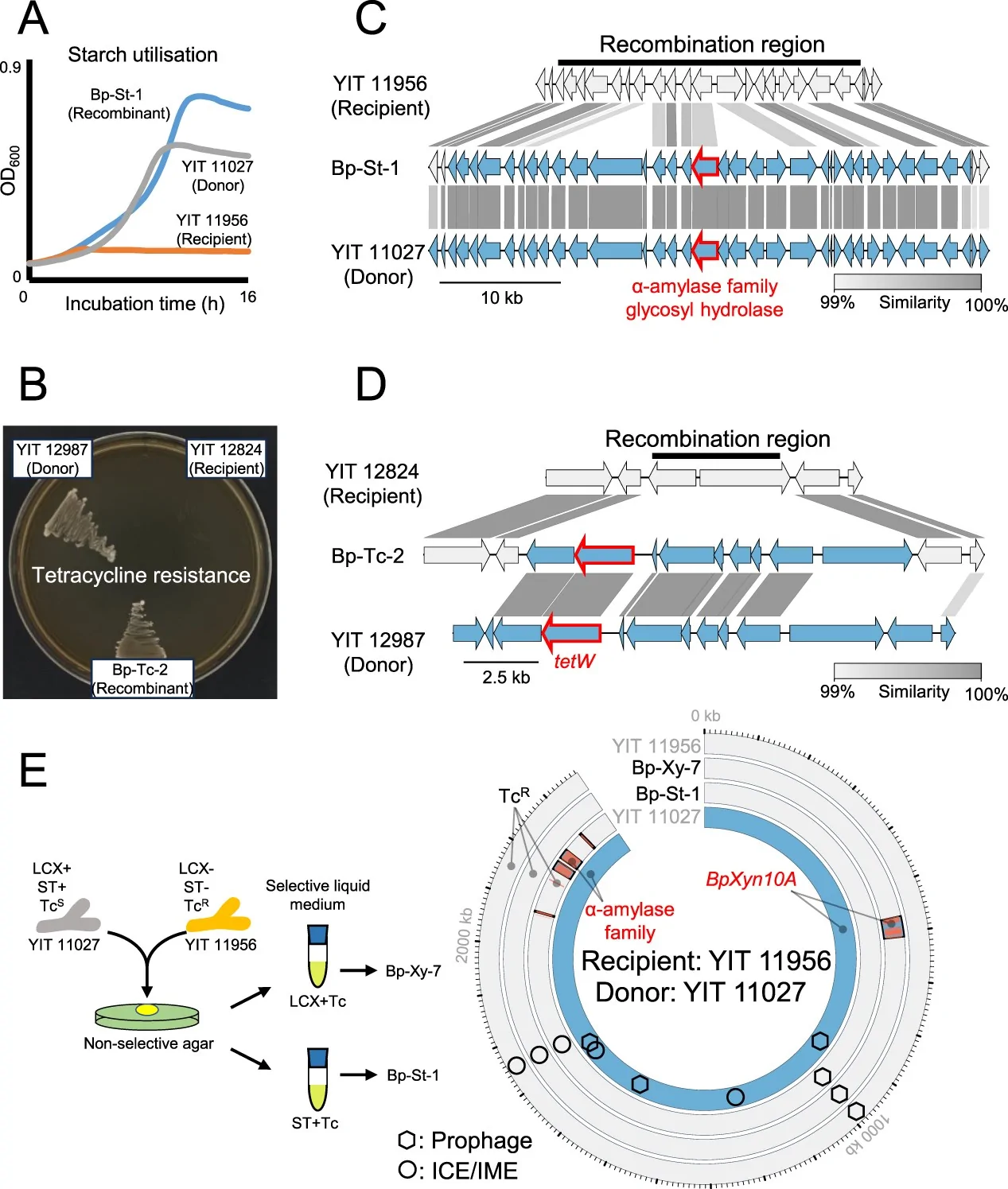
Horizontal transfer of multiple phenotypic traits via chromosomal recombination (A and B) phenotypic validation of recombinant strains. (A) Growth on starch as the sole carbon source are presented as growth curves (OD_600_), comparing the donor, recipient, and recombinant strains. (B) Colony formation on tetracycline-containing agar plates. **(**C and D) Donor-derived chromosomal segments identified in recombinant strains by whole-genome sequencing and fastGEAR analysis. (C) Donor-derived region harbouring an α-amylase family glycoside hydrolase gene in the starch-utilizing recombinant Bp-St-1 (recipient: YIT 11956; donor: YIT 11027). (D) Donor-derived region harbouring the tetracycline resistance gene *tetW* in the tetracycline-resistant recombinant Bp-Tc-2 (recipient: YIT 12824; donor: YIT 12987). Blue arrows: genes located within donor-derived chromosomal segments inferred by fastGEAR. Links between gene arrows: amino acid sequence similarity between proteins. (E) Representative chromosomal segments identified in recombinants derived from the same donor–recipient pair under selection for different phenotypic traits after co-culture for 16 h. “LCX+” and “LCX−”: presence and absence of the LCX utilization gene, respectively. “ST+” and “ST−”: starch utilization ability and inability, respectively. “S/R”: antibiotic sensitivity or resistance. See also Table S1 and Fig. S3 for all donor–recipient combinations and corresponding recombinants.

Chromosomal transfer could also proceed in both directions between co-cultured strains. After co-culturing YIT 12824 and YIT 4072^T^, subsequent selection yielded recombinants in both genetic backgrounds, with YIT 4072^T^ and YIT 12824 acquiring *BpXyn10A* and tetracycline-resistant gene (*tetW*), respectively (Table S1; Figs S2 and S3A).

These findings indicate that co-culture on agar can yield diverse recombinant strains carrying phenotype-linked chromosomal segments, enabling growth under conditions in which neither parental strain alone could thrive.

### Natural transformation mediates chromosomal transfer in *B. pseudocatenulatum*

To determine whether chromosomal transfer was mediated by donor-driven mechanisms (e.g. conjugation or transduction) or driven by recipient cells, we replaced living donor cells with heat-killed ones. Under this condition, tetracycline resistance was still horizontally transferred to the recipients. Whole-genome sequencing revealed that chromosomal recombination tracts in heat-killed and living donor-derived recombinants mainly overlapped (Figs 4 and S4), indicating that chromosomal transfer occurs via a recipient-driven process.

**Figure 4:**
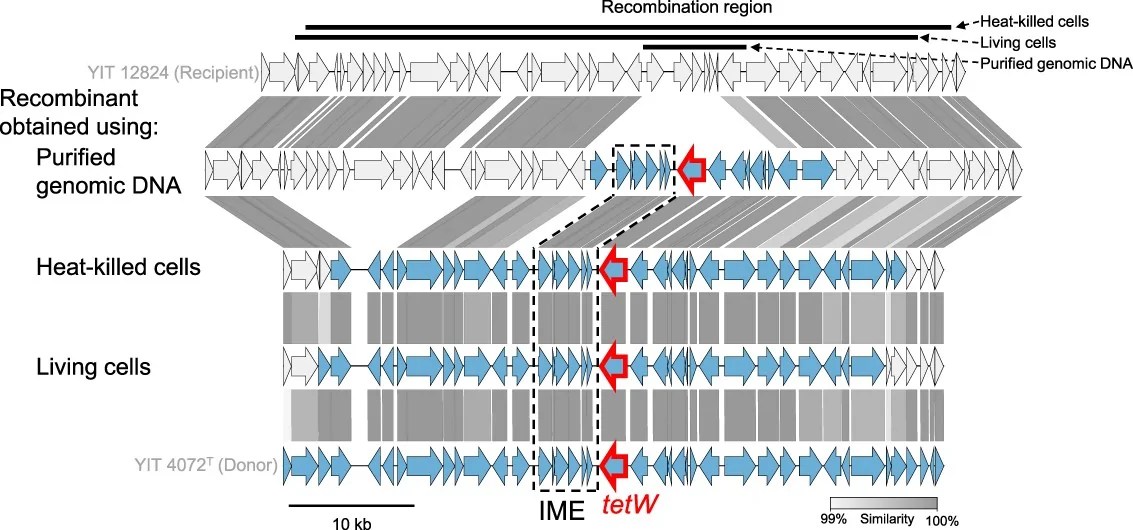
Natural transformation mediates chromosomal transfer in *B. pseudocatenulatum* chromosomal recombination tracts detected in recombinant strains obtained from YIT 4072^T^ and YIT 12824 as the recipient and donor, respectively, under three conditions: co-culture with living or heat-killed donor cells (Bp-Tc-5 or Bp-Tc-13, respectively), or supplementation with purified donor genomic DNA (Bp-Tc-11). The genomic positions of *tetW* and regions containing the IME are indicated. Blue arrows: fastGEAR-inferred genes encoded within donor-derived chromosomal segments. Links between gene arrows: amino acid sequence similarity between proteins.

Next, we tested whether extracellular DNA alone could mediate chromosomal transfer. The addition of purified donor genomic DNA to recipient cells induced the emergence of *tetW*-acquired strains (Figs 4 and S4). In addition, purified donor DNA also generated strains with acquired starch-utilization ability. In contrast, when DNase was added to co-cultures of living donor and recipient cells, no recombinant colonies were recovered. This result indicates that extracellular DNA is required for HGT under these experimental conditions, consistent with DNase-sensitive transformation rather than DNase-resistant HGT mechanisms such as conjugation or transduction [35]. These experiments position natural transformation as the mechanism responsible for chromosomal transfer in *B. pseudocatenulatum* under the tested conditions.

Specific chromosomal segments acquired following the addition of purified genomic DNA contained IMEs (Fig. 4), indicating that chromosomally encoded MGEs could also spread via natural transformation.

To assess whether natural transformation was restricted to a few laboratory-tested strains or was more broadly distributed, we examined 27 *B. pseudocatenulatum* isolates from our strain collection, listed in Table S2, for the acquisition of a *tetW*-carrying purified DNA fragment. Tetracycline-resistant transformants were detected in 12 of these isolates (Table S2), indicating that transformability is present in a substantial proportion of *B. pseudocatenulatum* strains. Natural transformation has been documented in only ~80 bacterial species, fewer than the number of species carrying phages or plasmids [36]. Our findings provide direct experimental evidence that natural transformation can occur in human gut bacteria under laboratory conditions.

### Chromosomal recombination generates macro- and micro-scale genomic variations

We quantified the extent of chromosomal recombination in 24 *B. pseudocatenulatum* recombinants derived from co-culture with living donor cells following 16 h of incubation on nonselective agar (Table S1). To evaluate the potential influence of donor–recipient sequence divergence on recombination-tract detection, we calculated pairwise SNP densities for the corresponding donor–recipient pairs. These pairs exhibited SNP densities from 4.17 to 8.18 SNPs/kb across the aligned genomic regions (Table S1), providing an estimate of the density of informative variants available for tract detection. Nevertheless, very short imported fragments may remain underdetected in genomic regions with low SNP density. Individual detected recombination events averaged 35.2 kb in length (range: 0.6–247.1 kb) (Fig. 5A). Each strain displayed an average of 2.1 recombination events (range: 1–6) (Fig. 5B). The cumulative donor-derived length was 73.2 kb per genome (range: 3.0–684.7 kb) (Fig. 5C), representing 3.0% (range: 0.12%–28.9%) of the recipient genome (Fig. 5D). The most extensively recombined strain, Bp-Xy-12, exhibited 28.9% replacement of its chromosome (Fig. 2B), approaching levels observed in eukaryotic meiosis [37], indicating that *B. pseudocatenulatum* could generate extensive genome-wide mosaicism through natural transformation. Some donor-derived chromosomal regions harboured genes commonly used for taxonomic and phylogenetic inference. In Bp-Xy-12, for example, these regions contained rRNA gene loci and several representative phylogenetic marker genes used for bifidobacterial classification [38] (Fig. S5). These observations indicate that chromosomal recombination can influence both phenotype-associated loci and genomic regions commonly used for phylogenetic inference.

**Figure 5:**
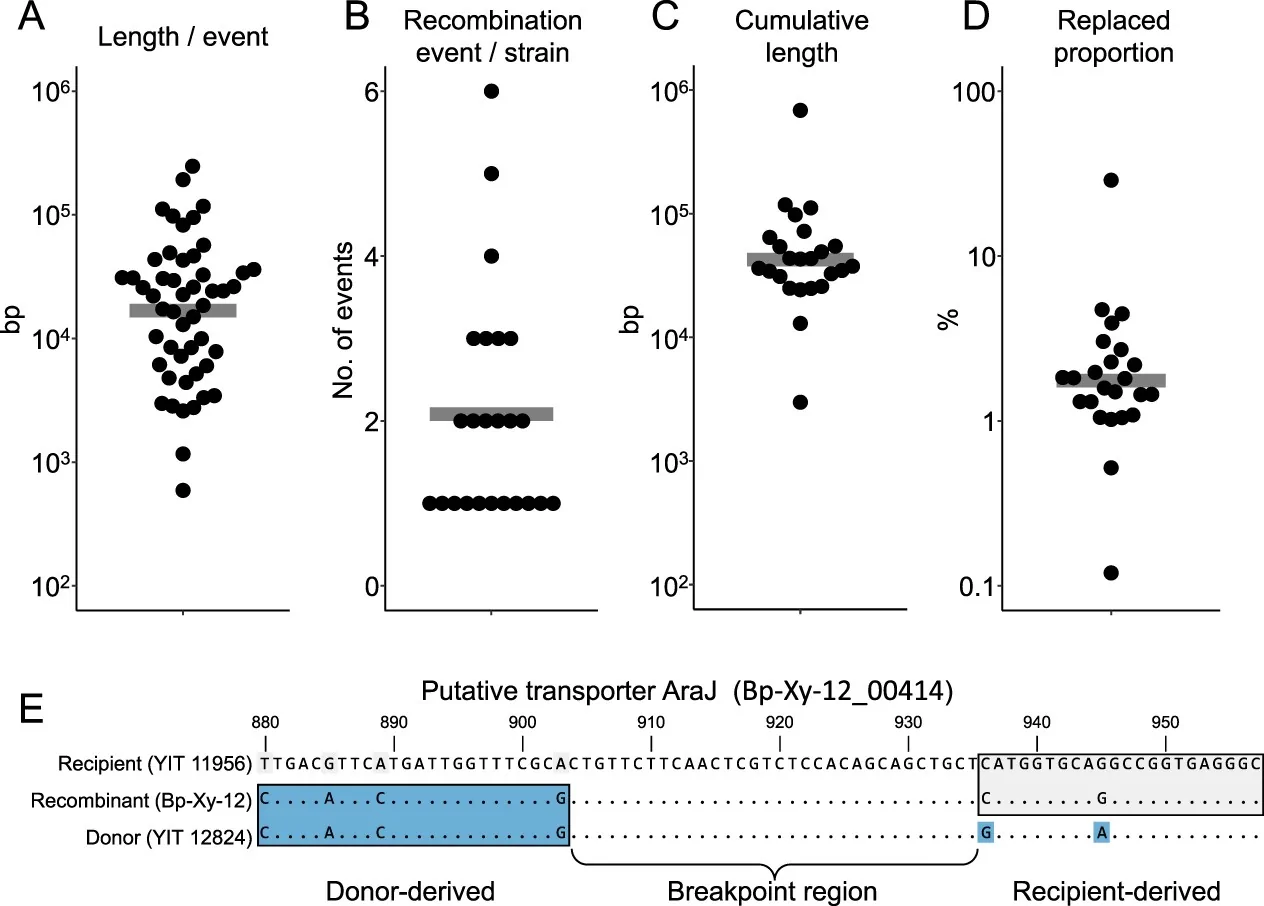
Extent and structure of chromosomal recombination *in vitro* (A–D) quantitative summary of chromosomal recombination in recombinant strains obtained after 16 h of co-culture with living donor cells (*n* = 24; see Table S1). Horizontal lines indicate mean values. (A) Length of individual recombination tracts. (B) Number of recombination events per strain. (C) Cumulative length of donor-derived chromosomal regions per recombinant. (D) Proportion of the recipient genome replaced by donor-derived segments. (E) Representative example of a recombination breakpoint located within a coding sequence, resulting in chimeric gene formation composed of donor- and recipient-derived sequences.

In addition to large chromosomal replacements, recombination breakpoints are occasionally located within coding sequences (Fig. 5E), generating chimeric genes composed of both donor and recipient-genome-derived sequences. Such intragenic recombination events introduce allelic diversity at the single-gene level, independently of gradual *de novo* mutation accumulation, indicating that natural transformation drives both large-scale genome restructuring and fine-scale sequence diversification, highlighting its capacity for creating genetic variation.

### Multiple environmental factors affect natural transformation frequency in *B. pseudocatenulatum*

The environmental cues that induce natural transformation differ among bacterial species and may reflect species-specific lifestyles [36]. This highlights the importance of defining environmental conditions that modulate natural transformation. We thus assessed conditions affecting transformation frequency in *B. pseudocatenulatum*.

First, we tested whether growth substrates modulate competence. Adding lactose, a representative fermentable sugar for bifidobacteria, reduced natural transformation frequency (Fig. 6A). We observed similar inhibitory effects with other fermentable carbohydrates (Fig. 6B), indicating that the presence of growth substrates suppresses natural transformation.

**Figure 6:**
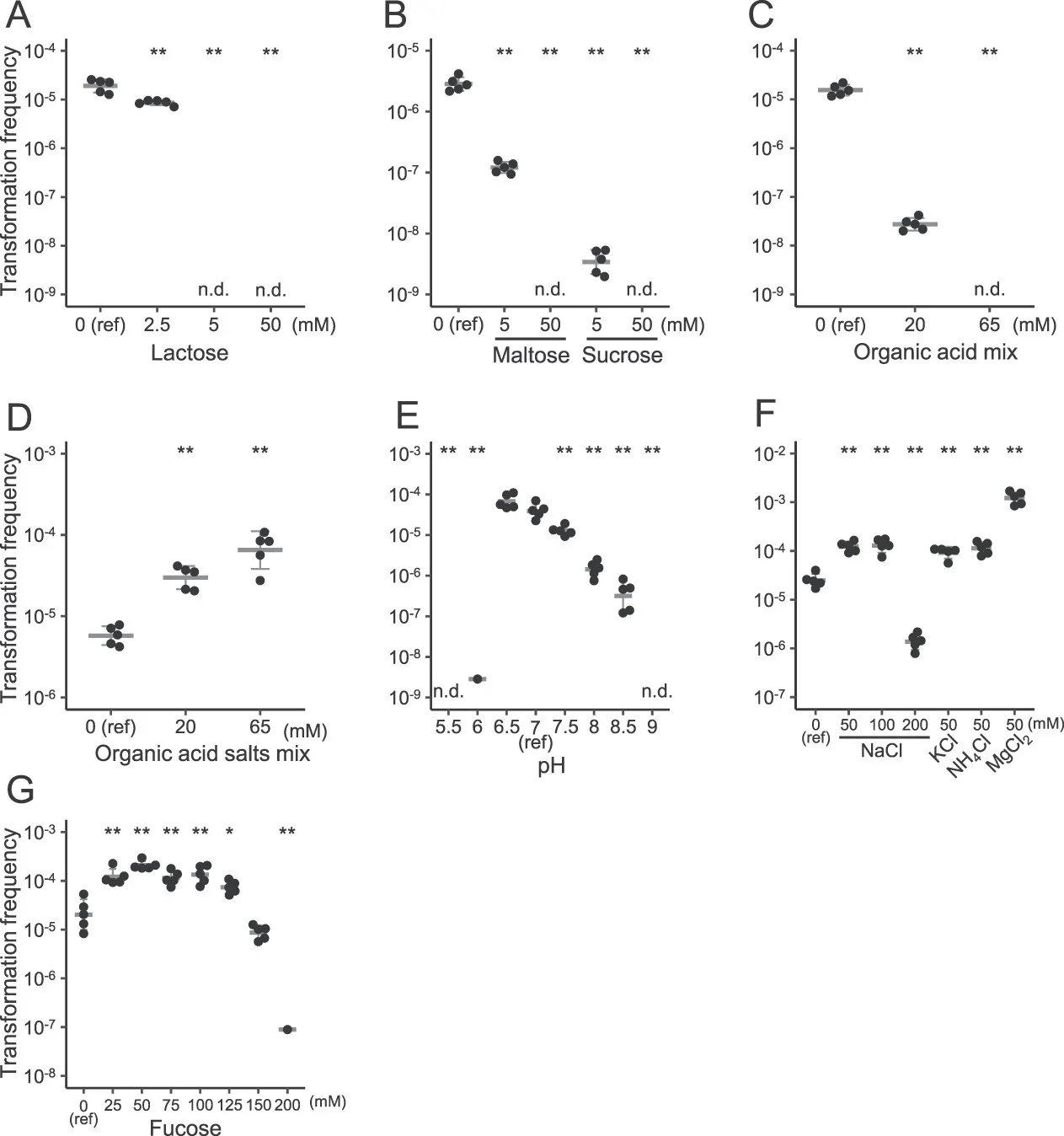
Environmental factors affecting natural transformation frequency in *B. pseudocatenulatum* natural transformation frequency measured by incubating the recipient strain YIT 12819 with purified *tetW*-carrying PCR fragments on mGAM agar under the indicated conditions. Effect of (A) lactose; (B) maltose and sucrose; (C) a mixture of organic acids (acetate, lactate, and formate); (D) a mixture of the corresponding sodium salts; (E) pH adjustment with hydrochloric acid or sodium hydroxide; (F) inorganic salts; (G) the non-fermentable sugar fucose. Each dot represents a biological replicate. Bars: mean ± standard deviation. n.d., not detected. In each panel, the reference condition is labelled “ref.” on the *x*-axis (A–D and F–G: 0 mM; E: pH 7.0). Asterisks: conditions that differed significantly from that of the corresponding reference (*: *P* < .05; **: *P* < .01; Mann–Whitney *U* test).

Next, as bifidobacteria ferment carbohydrates into organic acids (e.g. acetate, lactate, and formate) [39], we evaluated the impact of these metabolites. Supplementation with these organic acids significantly reduced transformation frequency (Fig. 6C). In contrast, sodium salts of the same acids exerted no inhibitory effect (Fig. 6D), indicating that protons, rather than organic acid anions, mediate suppression. Consistent with this interpretation, lowering the pH with hydrochloric acid strongly inhibited transformation at pH ≤6.0, with transformation frequency peaking at pH 6.5 (Fig. 6E). As the typical human colonic pH range is 5.5–7.5 [40], these results position proton concentration as a potential ecological cue regulating natural transformation in the human gut.

Sodium salts of organic acids enhanced transformation frequency despite unchanged pH (Fig. 6D), suggesting the involvement of additional ionic or osmotic effects. Consistently, supplementation with sodium chloride promoted transformation at concentrations of ≤100 mM but inhibited it at 200 mM (Fig. 6F). Similarly, potassium, ammonium, and magnesium chloride at 50 mM increased transformation frequency (Fig. 6F). Furthermore, the non-fermentable sugar fucose enhanced transformation within a specific concentration range (Fig. 6G). These observations indicate that soluble compounds modulate natural transformation in a concentration-dependent manner. These compounds could influence recipient cells and/or extracellular donor DNA molecules through ionic or osmotic effects.

In summary, natural transformation in *B. pseudocatenulatum* is regulated by fermentation-associated and ionic conditions, suggesting coordination with cellular metabolic state.

### Identification of genes essential for natural transformation using reverse genetics

To identify genes involved in natural transformation in *B. pseudocatenulatum*, we compared the transcriptional profiles between transformation-promoting and suppressing conditions (i.e. 50 mM MgCl_2_ supplementation and pH 6.0, respectively) using RNA sequencing. Several genes showed >10-fold increase in expression upon promoted transformation (Table S3).

Next, we established a natural transformation-based markerless genome editing system, adapted from methods developed for *Bacillus* [32], to assess the functional roles of genes using reverse genetics. This approach enables genome editing by introducing linear DNA constructs into recipient cells under transformation-promoting conditions, thereby avoiding the use of plasmid vectors, electroporation, and subsequent plasmid-curing steps.

We generated deletion mutants of five individual genes and three gene clusters identified by transcriptomic analysis (Table S3). Natural transformation was completely abolished in strains lacking any of the following: tight adherence (Tad) pili gene cluster (YIT12819_00143-00149), DNA binding protein ComEA/channel protein ComEC gene cluster (YIT12819_00905-00906), or DNA processing protein DprA/DNA recombination helicase ComM/putative endonuclease YraN gene cluster (YIT12819_00675-00677), suggesting that these gene clusters are essential for natural transformation (Fig. 7A). Because deletion of the DprA–ComM–YraN gene cluster did not distinguish the contribution of each individual gene, we further examined individual deletion mutants of *dprA*, *comM*, and *yraN*. As observed for the cluster deletion mutant, no transformants were recovered from any of these single-gene deletion mutants, suggesting that each of these genes is required for natural transformation. In contrast, deletion of other candidate genes did not significantly affect transformation frequency (Fig. 7B).

**Figure 7:**
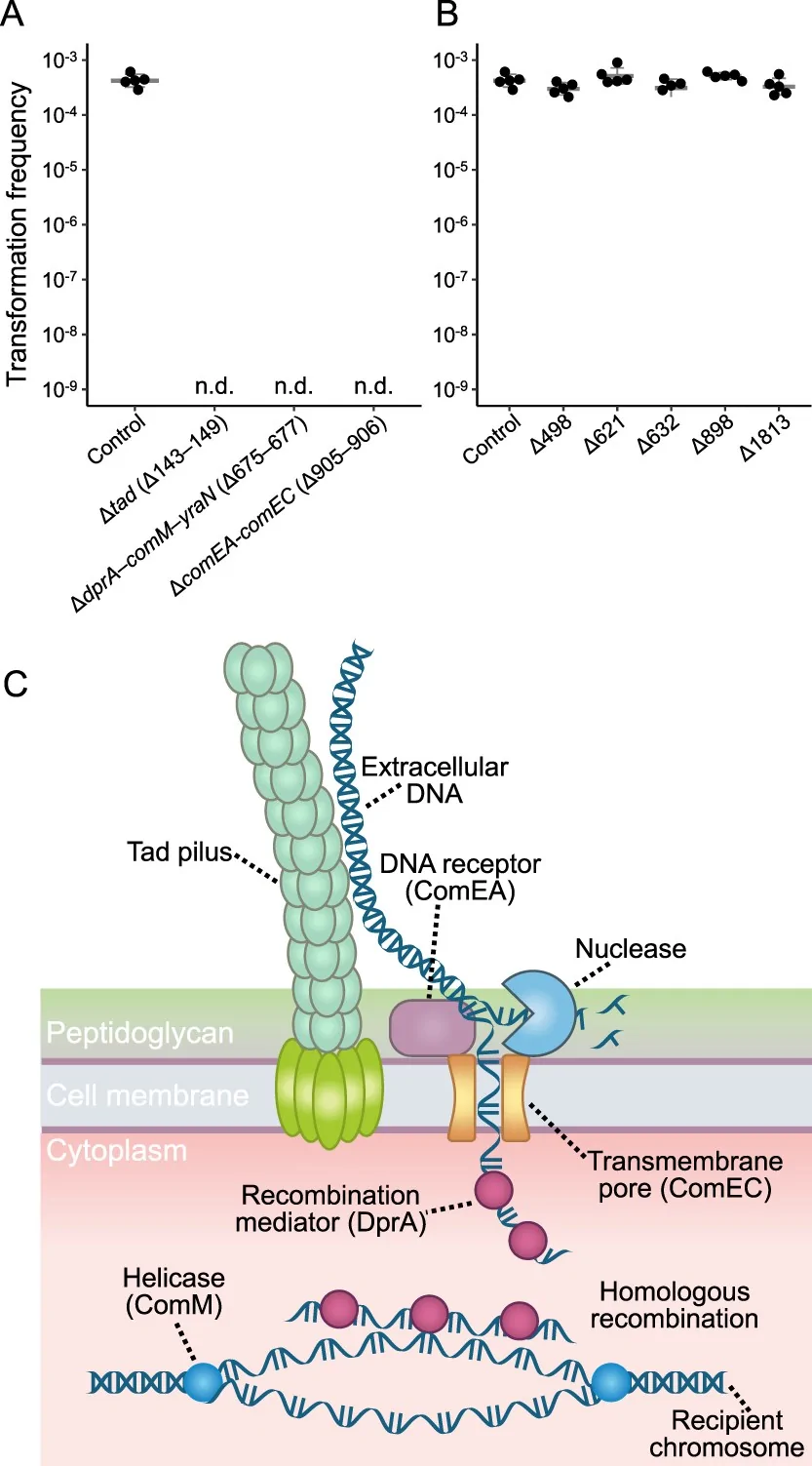
Genetic requirements for natural transformation identified by reverse genetics in *B. pseudocatenulatum* (A) transformation frequency of mutants with abolished transformation, including those lacking the Tad pili genes (Δ143–149), *comEA*–*comEC* (Δ905–906), or *dprA*–*comM*–*yraN* (Δ675–677) clusters. (B) Transformation frequency of mutants with no measurable effect on transformation frequency, including those lacking genes encoding a single-strand DNA-binding protein (Δ498), PGAP1-like protein (Δ621), WXG100 family protein (Δ632), a protein of unknown function (Δ898), or an LPXTG-motif cell wall-anchored protein (Δ1813). Deletion mutants are labelled by the last three digits of the locus tag (e.g. Δ498 = ΔYIT12819_00498). Ranges indicate cluster deletions (e.g. Δ143–149 = ΔYIT12819_00143–00149). (C) Schematic model of natural transformation in *B. pseudocatenulatum*, based on predicted gene functions and established mechanisms in Gram-positive bacteria. Extracellular DNA is captured by a Tad-associated apparatus, delivered to the DNA receptor ComEA, translocated through ComEC, processed by DprA, and integrated into the chromosome via RecA-mediated homologous recombination, with ComM facilitating recombination.

Together with the predicted functions of these genes and established mechanisms of natural transformation in Gram-positive bacteria, these results support a schematic model. In this model, extracellular DNA is captured by the Tad-associated apparatus, delivered to the DNA receptor ComEA, translocated through ComEC, processed by DprA, and integrated into the chromosome via RecA-mediated homologous recombination, with ComM facilitating the recombination process (Fig. 7C) [36, 41].

### Natural transformation occurs in multiple bifidobacterial species

To determine whether natural transformation extends in other members of the genus *Bifidobacterium*, we first examined the distribution of essential gene clusters for natural transformation across representative bifidobacterial species. Most species exhibited conserved Tad genes, *comEA–comEC*, and *dprA–comM–yraN* cluster homologs (Fig. 8A), suggesting that the capacity for natural transformation may be widely distributed across the genus.

**Figure 8:**
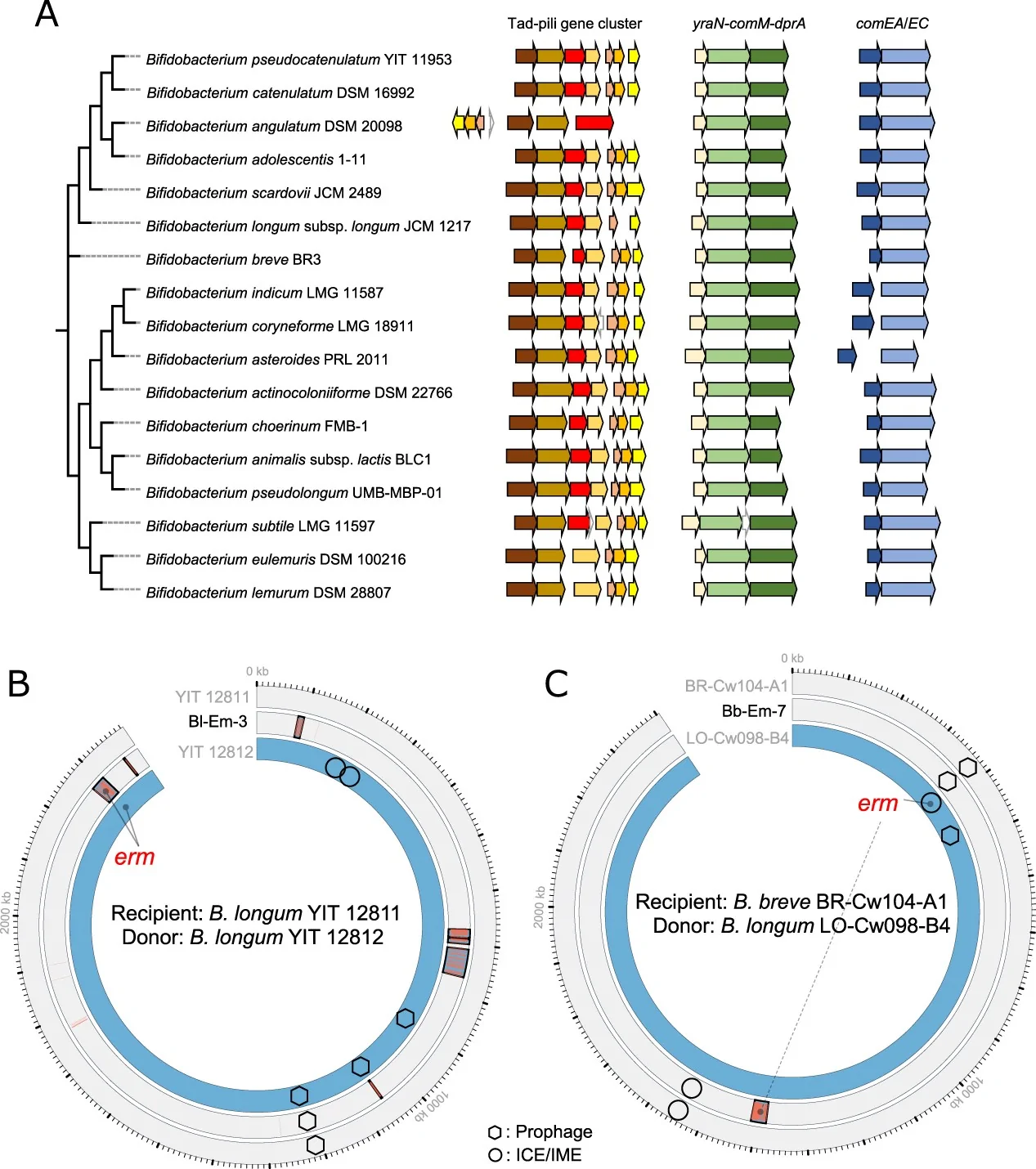
Conservation of natural transformation capacity in bifidobacteria (A) conservation of three gene clusters essential for natural transformation across representative bifidobacterial species. Same colour: genes sharing the same predicted function. (B and C) Recombination events detected in *B. longum* subsp. *longum* and *B. breve* strains, revealing the horizontal transfer of the erythromycin resistance gene (*erm*) via chromosomal recombination. Hexagons and circles: phage and ICE/IME insertion sites, respectively. See also Table S1 and Fig. S6 for all donor–recipient combinations and corresponding recombinants.

Next, we tested whether natural transformation could occur under laboratory conditions in two other human-associated species, *B. longum* and *B. breve*. In both species, the erythromycin resistance gene (*erm*) was horizontally transferred when we mixed donor and recipient strains on agar, resulting in mosaic genomes generated through chromosomal recombination (Figs 8B, S6A, and  B; Table S1). Using *B. breve* as a representative species, we obtained erythromycin-resistant recombinants upon supplementation with heat-killed donor cells or purified genomic DNA, consistent with natural transformation as a transfer mechanism.

Transfer of *erm* from *B. longum* donor to *B. breve* recipient demonstrated that natural transformation could mediate HGT across species (Figs 8C and S6B), consistent with suggestions that *B. breve* could acquire chromosomal genes from *B. longum* [42].

Taken together, our findings indicate that natural transformation is not restricted to *B. pseudocatenulatum* but represents a conserved mechanism within bifidobacteria, enabling chromosomal gene transfer both within and between species.

## Discussion

### Natural transformation as a driver of genome plasticity in bifidobacteria

In human gut bacteria, recombination can transfer adaptive alleles across lineages [43]. However, the mechanisms and ecological contexts enabling large-scale chromosomal transfer in gut commensals remain largely unknown. In this study, we demonstrated that natural transformation could rapidly generate mosaic chromosomes in human-associated bifidobacteria. Within a single overnight incubation, distinct lineage-derived segments replaced multiple recipient chromosomal regions, with individual imports reaching up to 247 kb and cumulative replacements accounting for 28.9% of the chromosome (Fig. 5). Although the *in vivo* chromosomal transfer between coexisting lineages cannot be readily explained by plasmid- or phage-mediated transfer (Fig. 1), our *in vitro* experiments demonstrated that natural transformation provides a mechanistic explanation for these events. These findings offer a mechanistic basis for the increased homologous recombination rates previously reported in bifidobacteria [12], suggesting that natural transformation might represent an important mechanism underlying strain-level genome plasticity in this genus. The scale of recombination tracts we observed is notable among naturally transformable bacteria. In well-studied competent species (e.g. *Helicobacter pylori*), recombination tracts typically follow a bimodal distribution centred at ~28 bp and ~1.6 kb [44], whereas *Vibrio cholerae* acquires DNA fragments exceeding 150 kb via natural transformation [45]. The chromosomal imports detected in *B. pseudocatenulatum* exceed those typically observed in *H. pylori* and approach the upper range reported for *V. cholerae*, positioning bifidobacteria among bacterial taxa capable of large-scale chromosomal transfer through natural transformation.

### Ecological and evolutionary implications for rapid adaptation

Our experiments demonstrated that natural transformation can generate phenotypic innovations, enabling growth under conditions in which neither parental strain alone could thrive. Ecologically, natural transformation accelerates the generation of new gene combinations, subsequently filtered by environmental selection. This process could alter patterns of resource use and promote niche expansion. Because carbohydrate metabolism is crucial in bifidobacterial ecology [46], the acquisition of CAZyme-encoding chromosomal regions broadens or fine-tunes dietary substrate utilization across host-associated niches.

We found that some donor-derived chromosomal regions harboured rRNA gene loci and representative phylogenetic marker genes used for bifidobacterial classification. Therefore, chromosomal recombination should be considered when interpreting phylogenetic relationships, strain classification, and population genomic analyses in bifidobacteria.

### Mechanistic insights and the genetic basis of natural transformation

A combined transcriptomic profiling and reverse genetics enabled the identification of Tad pili, the ComEA–ComEC DNA uptake channel, and the DprA–ComM–YraN processing module as essential natural transformation components in *B. pseudocatenulatum*. These components correspond to the canonical models of natural transformation in Gram-positive bacteria, encompassing DNA capture at the cell surface, conversion to single-strand DNA, translocation across the membrane, and RecA-mediated homologous recombination (Fig. 7C). Tad machinery disruption completely abolished transformation, revealing a previously unrecognized role of Tad pili in addition to host adhesion in this taxon [47]. Conservation of these gene clusters across the genus, together with experimental demonstration in *B. longum* and *B. breve* (Fig. 8), indicates likely widespread natural transformation capacity among bifidobacteria.

### Ecological regulation and practical applications

Natural transformation is regulated by habitat-specific cues in diverse environments, including chitin-associated aquatic habitats and nutrient-limited environments [48]. Similarly, natural transformation in *B. pseudocatenulatum* is not constitutive but is modulated by environmental conditions. Such environmental regulation may account for the fact that this phenomenon remained undetected for nearly a century of bifidobacterial research: conventional bifidobacteria-specific, typically fermentable carbohydrate-enriched media suppress transformation activity (Fig. 6A and B). The widespread use of these culture conditions in laboratory studies has therefore likely masked transformation events.

In addition to carbohydrate availability, transformation frequency was strongly inhibited at pH ≤6.0, reaching a maximum near pH 6.5, and was further modulated in a concentration-dependent manner by soluble compounds, including non-fermentable sugars (Fig. 6). As bifidobacteria acidify their surroundings by fermenting carbohydrates, these observations suggested a self-regulatory logic: resource-rich conditions, that promote acid production, favour genome stability, whereas resource-limited conditions, maintaining a more neutral pH, promote genetic diversification through natural transformation. This model links metabolic state to genome plasticity, providing a biologically plausible ecological context for when and where natural transformation might occur in the human gut.

From a practical perspective, our results expand the genetic toolbox available for bifidobacteria. Genetic manipulation in this genus has historically been challenging, as DNA delivery is often hindered by restriction-modification systems and strong strain-specific variation in transformability [49]. Although substantial progress has been achieved through the methylation-assisted bypass of restriction barriers [50] and the development of clustered regularly interspaced short palindromic repeat (CRISPR)-based tools [51], these approaches still typically rely on plasmid introduction, extensive optimization, and subsequent plasmid curing. In contrast, the herein-described natural transformation-based strategy exploits an endogenous DNA-uptake machinery to introduce linear DNA directly into recipient cells, enabling plasmid-free and markerless genome editing under transformation-promoting conditions (Fig. S7). This framework could facilitate functional genetic analysis in *B. pseudocatenulatum* and be extended to a broader range of bifidobacterial species.

### Limitations and perspectives

The gut environment differs substantially from the conditions we used in the experiment (e.g. continuous flow, mucus layers, host immune factors, and complex microbial community interactions). These features likely influence DNA availability, competence regulation, and selective pressure on recombinant cells. Future studies should assess transformation dynamics *in vivo* across different dietary regimes, age groups, and health states, quantify DNA flux and competence windows, and determine the genomic distribution of recombination events within complex communities. Such investigations will be essential for defining the spatiotemporal contexts of natural transformation in the gut and for assessing its contribution to *in situ* adaptive evolution.

## Supplementary Material

Supplementary_information_wrag208

Supplementary_tables_wrag208

## Data Availability

Genome sequence and raw RNA sequencing data are deposited in the NCBI database under BioProject ID PRJNA1033448.
