# Supplementary material for: Natural transformation drives large-scale genome mosaicism in human gut bifidobacteria
Source: ISME J. 2026 Aug 8;20(1):wrag208. doi: 10.1093/ismejo/wrag208 (PMC13660472; doi:10.1093/ismejo/wrag208)
Supplement: Supplementary file 1 [file supplementary_information_wrag208.pdf]

Supplemental information

Supplementary methods

Detailed procedure of long-arm donor DNA preparation

tetW was PCR-amplified from the genome of strain YIT 4072^T. The upstream and downstream homologous arms flanking the selected intergenic region of the YIT 12819 chromosome were amplified separately and were 1,018 bp and 1,886 bp in length, respectively. These three fragments were joined by overlap-extension PCR (OE-PCR) using KOD-Plus Neo DNA polymerase (Toyobo, Osaka, Japan), yielding a 5,462 bp linear construct. The purified construct was mixed with YIT 12819 cells and spotted onto mGAM agar supplemented with 50 mM $MgCl_2$. After overnight anaerobic incubation, the cells were recovered and plated onto tetracycline-containing selective agar. Single colonies were isolated, and one specific verified isolate was designated YIT 12819Tc. Next, high-molecular-weight genomic DNA extracted from YIT 12819Tc was used as the template to amplify a long-arm donor DNA. The primers used for amplification are listed in Table S5.

The overall workflow of markerless genome manipulation

A schematic overview of the natural transformation-based markerless genome-editing workflow is shown in Figure S7.

Details of *upp* deletion mutant construction

The 3.4 kb upstream and 3.5 kb downstream flanking regions of the *upp* locus in YIT 12819 were PCR-amplified using KOD-Plus Neo DNA polymerase. The two fragments were OE-PCR-joined to generate a 6.9 kb linear deletion construct. The purified construct was mixed with YIT 12819 cells and spotted onto mGAM agar supplemented with 50 mM $MgCl_2$. After overnight anaerobic incubation, the cells were recovered and plated onto 100-µg/mL 5-fluorouracil (5FU)-containing mGAML agar, and 5FU-resistant colonies were screened by colony PCR and Sanger sequencing. A strain in which the *upp* locus was successfully deleted was designated YIT 12819Δ*upp* and used for subsequent experiments. The primers used for the amplification are listed in Table S6.

Details of *the upp–tetW* counterselection cassette preparation

To incorporate the strain YIT 4072^T-derived *tetW* gene into an intergenic region located

downstream of *upp* in the YIT 12819 genome, a 5.7 kb linear construct was OE-PCR-assembled. The purified construct was mixed with YIT 12819 cells and spotted onto mGAM agar supplemented with 50 mM $MgCl_2$, followed by overnight anaerobic incubation. The resulting cells were subsequently plated onto tetracycline-containing mGAML agar, and tetracycline-resistant colonies were isolated, which carried a chromosomal configuration with tandem-positioned *upp* and *tetW* at the targeted locus. Finally, genomic DNA extracted from one verified isolate was used as a template to amplify a 3.6 kb linear *upp–tetW* cassette for subsequent genome engineering steps. The primers used to construct this cassette are listed in Table S7.

Details of Markerless deletion of target genes

The 1–2 kb genomic regions located upstream and downstream of the target gene were PCR-amplified using KOD-Plus Neo DNA polymerase. The upstream homologous arm, *upp–tetW* cassette, and downstream arm (engineered to contain a 60 bp direct-repeat sequence identical to the terminal portion of the upstream arm) were OE-PCR-assembled, generating a linear construct of approximately 6–7 kb. The purified construct was mixed with YIT 12819Δ*upp* cells and spotted onto mGAM agar supplemented with 50 mM $MgCl_2$. After overnight anaerobic incubation, the cells were recovered and plated onto 10 µg/mL tetracycline-containing mGAML agar to isolate strains with an integrated construct at the target locus.

Next, insertion strains were cultured in mGAML broth to the exponential growth phase and plated onto 100 µg/mL 5-fluorouracil (5FU)-containing mGAML agar. Under these counterselection conditions, homologous recombination between the direct-repeat sequences excised the *upp–tetW* cassette, yielding 5FU-resistant colonies. Colonies lacking both the target gene and the *upp–tetW* cassette were colony PCR-identified and subsequently validated using Sanger sequencing. Strains confirmed to carry the expected deletion genotype were designated as markerless deletion mutants. The primers used for the construction of natural transformation-defective deletion mutants are listed in Table S8.

Supplementary figures

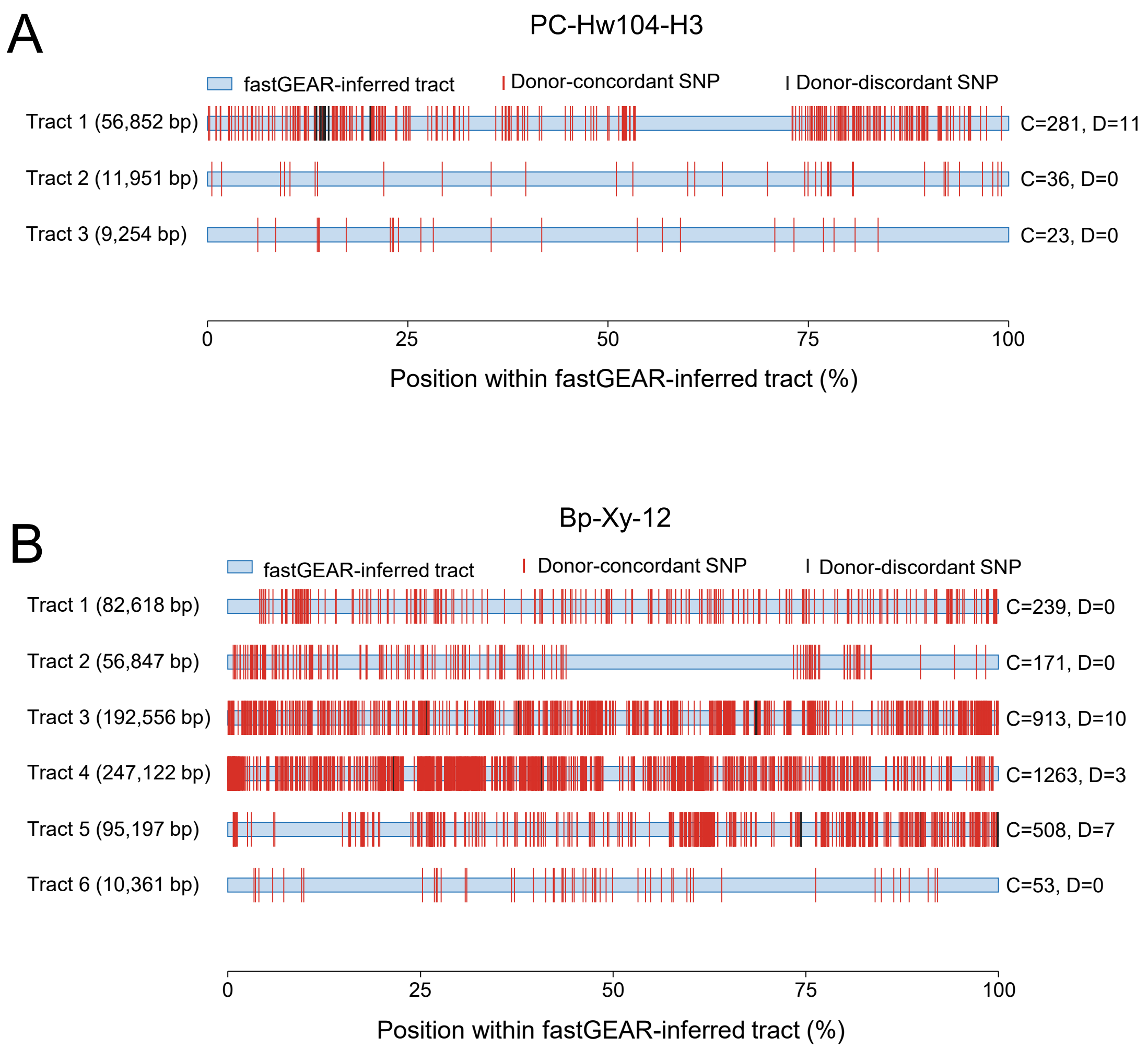

Figure S1. Donor-concordant and donor-discordant SNPs within fastGEAR-inferred recombination tracts

The distributions of SNPs within representative fastGEAR-inferred recombination tracts are shown for the infant-derived recombinant presented in Figure 1 (**A**) and the *in vitro*-derived recombinant presented in Figure 2 (**B**). Donor-concordant SNPs indicate positions at which the recombinant and donor share the same allele that differs from the recipient allele, whereas donor-discordant SNPs indicate positions within the inferred recombination tract at which the recombinant allele does not match the donor allele.

Alt text: Donor-concordant SNPs cluster within inferred recombination tracts, whereas donor-discordant SNPs are sparse and scattered.

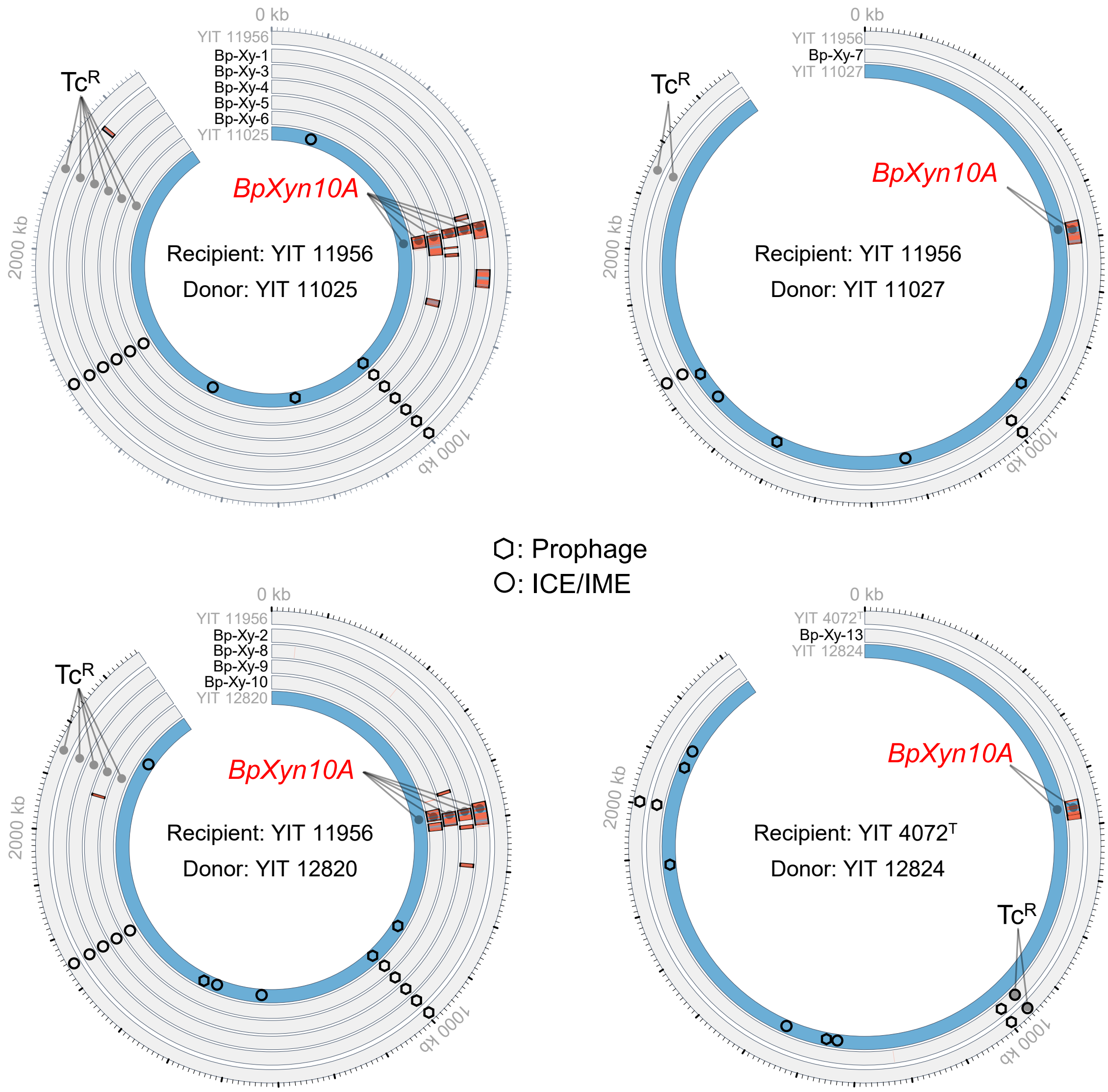

Figure S2. LCX utilisation-associated donor-derived chromosomal segments

Genome-wide recombination events in recombinant *Bifidobacterium pseudocatenulatum* strains that acquired LCX utilisation. For each donor–recipient pairing, Circos plots display the recipient chromosome (outer circle) and recombinant genomes (inner circles). Blue shaded segments in recombinants: fastGEAR-inferred donor-derived chromosomal tracts. Red lines: donor-concordant SNP. Hexagons and circles: prophage and ICE/IME integration sites, respectively.

Alt text: Circular genome maps show donor-derived chromosomal segments in recombinant strains selected for xylan utilisation.

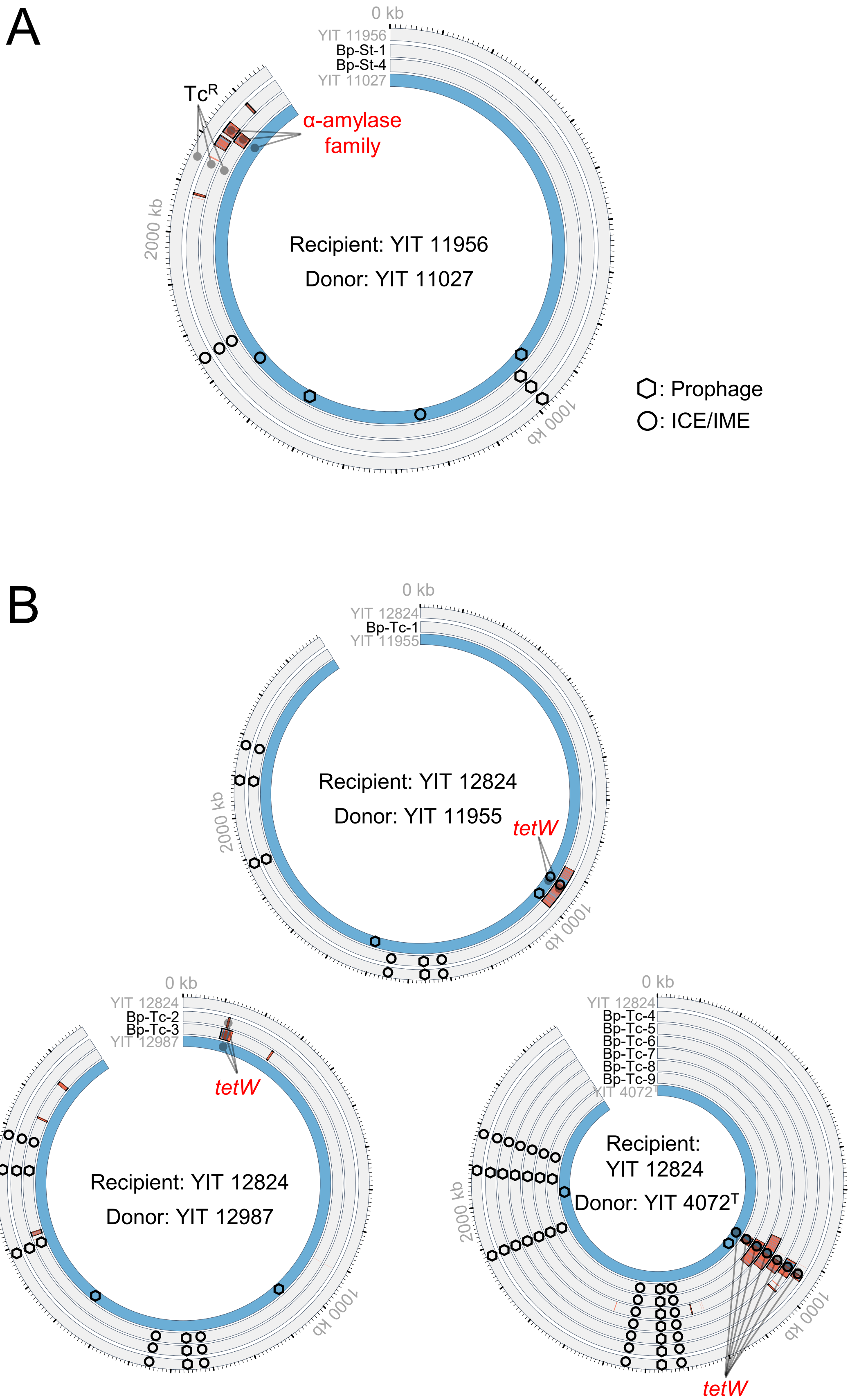
A
0 kb
YIT 11956
Bp-St-1
Bp-St-4
YIT 11027
TcR
α-amylase
family
2000 kb
1000 kb
Recipient: YIT 11956
Donor: YIT 11027
: Prophage
: ICE/IME
B
0 kb
YIT 12824
Bp-Tc-1
YIT 11955
Recipient: YIT 12824
Donor: YIT 11955
tetW
2000 kb
1000 kb
0 kb
YIT 12824
Bp-Tc-2
Bp-Tc-3
YIT 12987
tetW
Recipient: YIT 12824
Donor: YIT 12987
2000 kb
1000 kb
0 kb
YIT 12824
Bp-Tc-4
Bp-Tc-5
Bp-Tc-6
Bp-Tc-7
Bp-Tc-8
Bp-Tc-9
YIT 4072T
Recipient:
YIT 12824
Donor: YIT 4072T
2000 kb
1000 kb
tetW

Figure S3. Starch utilisation- or tetracycline resistance-associated donor-derived chromosomal segments

Genome-wide recombination events detected in recombinant *Bifidobacterium pseudocatenulatum* strains that acquired either **(A)** starch utilisation or **(B)** tetracycline resistance through chromosomal recombination. For each donor–recipient pairing, Circos plots display the recipient chromosome (outer circle) and recombinant genomes (inner circles). Blue shaded segments in recombinants: fastGEAR-inferred donor-derived chromosomal tracts. Red lines: donor-concordant SNPs. Hexagons and circles: prophage and ICE/IME integration sites, respectively.

Alt text: Circular genome maps show donor-derived chromosomal segments in recombinant strains selected for starch utilisation or tetracycline resistance.

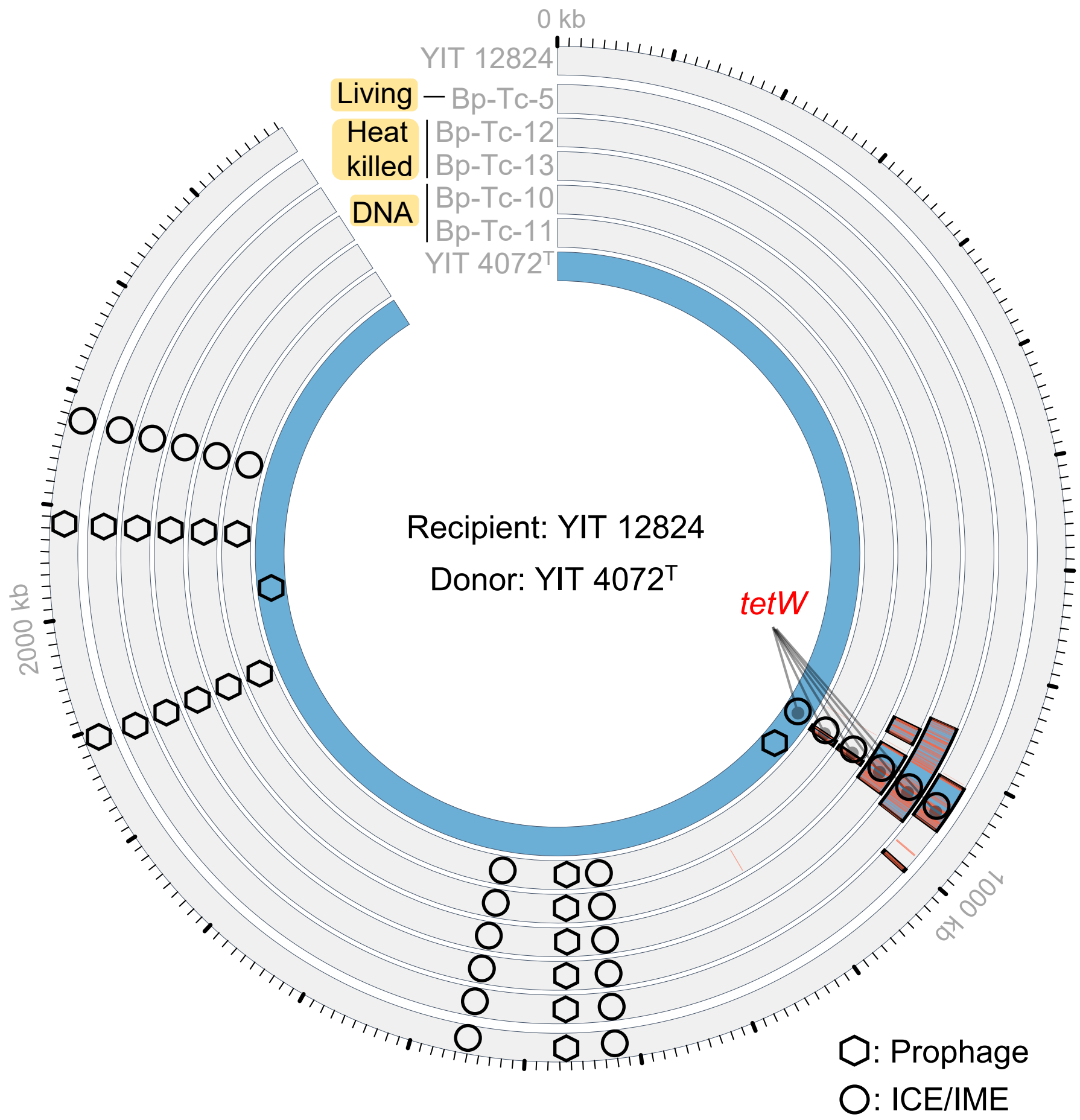

Figure S4. Chromosomal recombination following exposure to living donors, heat-killed donors, or purified genomic DNA

Illustration of genome-wide recombination events detected in YIT 12824 when exposed to living donor cells, heat-killed donor cells, or purified donor genomic DNA, demonstrating that chromosomal transfer occurs independently of donor viability. Circos plots display the recipient chromosome (outer circle) and the corresponding recombinant genomes (inner circles). Blue shaded segments in recombinants: fastGEAR-inferred donor-derived chromosomal tracts. Red lines: donor-concordant SNPs. Hexagons and circles: prophage and ICE/IME element integration sites, respectively.

Alt text: Genome-wide maps show similar donor-derived chromosomal segments in recipient strains exposed to live cells, heat-killed cells, or purified DNA, indicating transfer independent of donor viability.

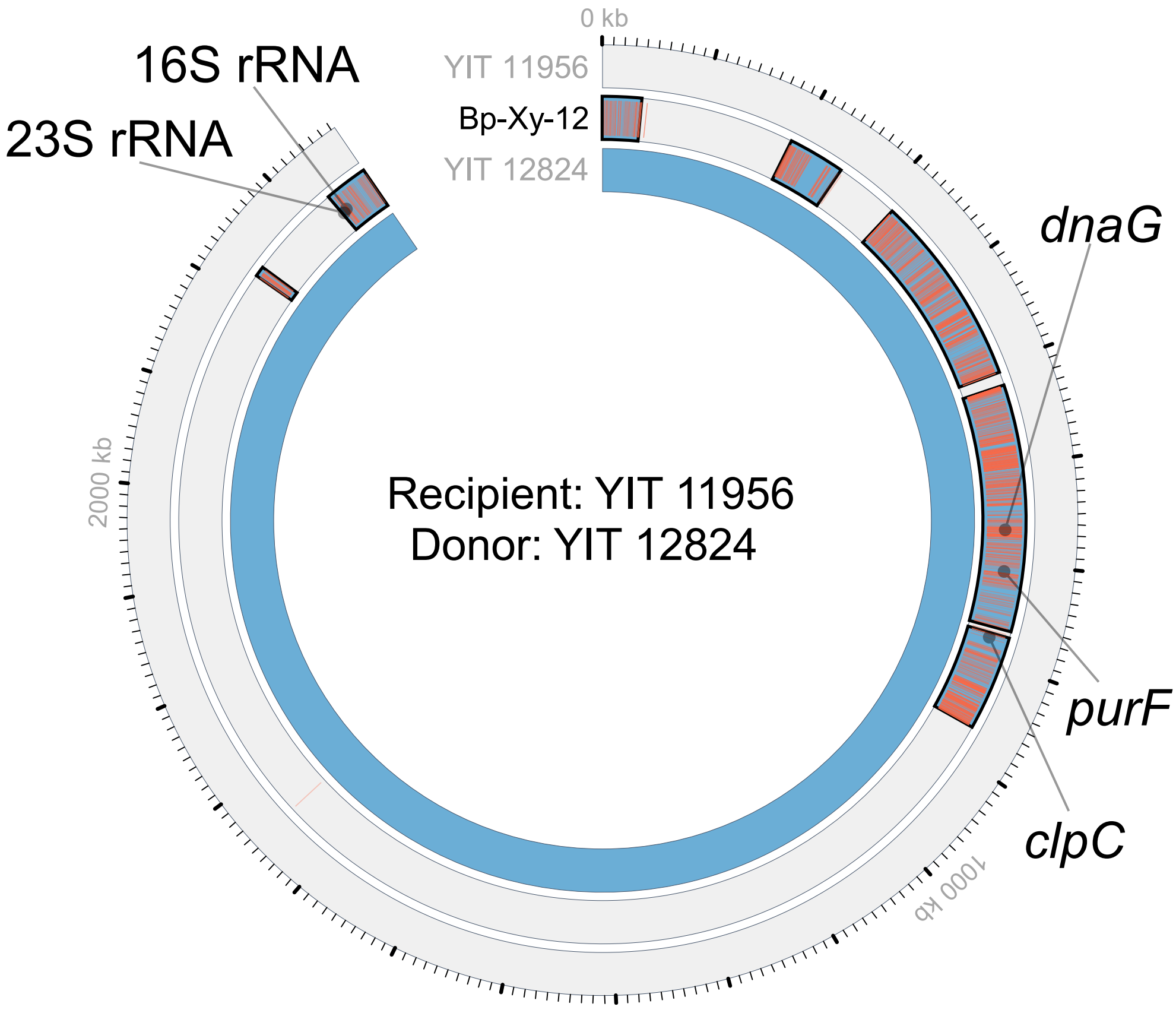

Figure S5. Donor-derived recombination tracts harbouring representative phylogenetic marker genes

Genome-wide recombination events detected in Bp-Xy-12, the recombinant strain with the largest proportion of donor-derived chromosomal replacement. Circos plots display the recipient chromosome (outer circle) and the corresponding recombinant genomes (inner circles). Blue shaded segments in recombinants: fastGEAR-inferred donor-derived chromosomal tracts. Red lines: donor-concordant SNPs. Blue shaded segments indicate donor-derived chromosomal regions inferred by fastGEAR. Lines terminating in filled circles indicate the genomic positions of representative phylogenetic marker genes used for bifidobacterial classification [38].

Alt text: Genome-wide maps show donor-derived recombination tracts in Bp-Xy-12, with marker gene positions indicated by lines, illustrating that representative phylogenetic marker genes are located within acquired chromosomal regions.

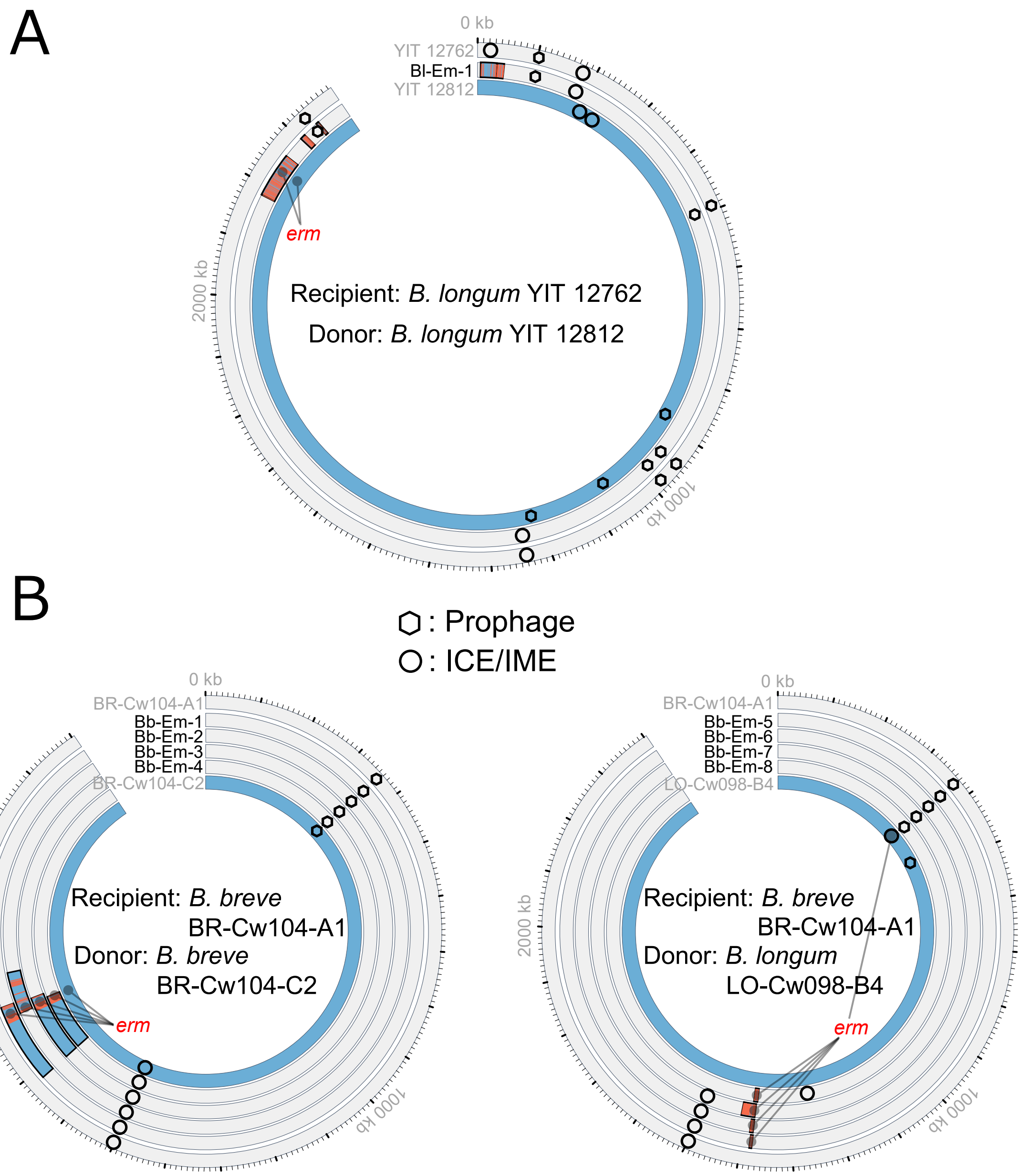

Figure S6. Chromosomal recombination in *Bifidobacterium longum* subsp. *longum* and *B. breve*

Donor-derived chromosomal segments acquired via natural transformation in recombinant strains of **(A)** *B. longum* subsp. *longum* and **(B)** *B. breve*. Circos plots display the recipient chromosome (outer circle) and recombinant genomes (inner circles). Blue shaded segments in recombinants: fastGEAR-inferred donor-derived tracts. Red lines: donor-concordant SNPs. Hexagons and circles: prophage and ICE/IME integration sites, respectively.

Alt text: Circular genome maps show donor-derived chromosomal segments in *Bifidobacterium longum* and *Bifidobacterium breve* recombinants selected for erythromycin resistance.

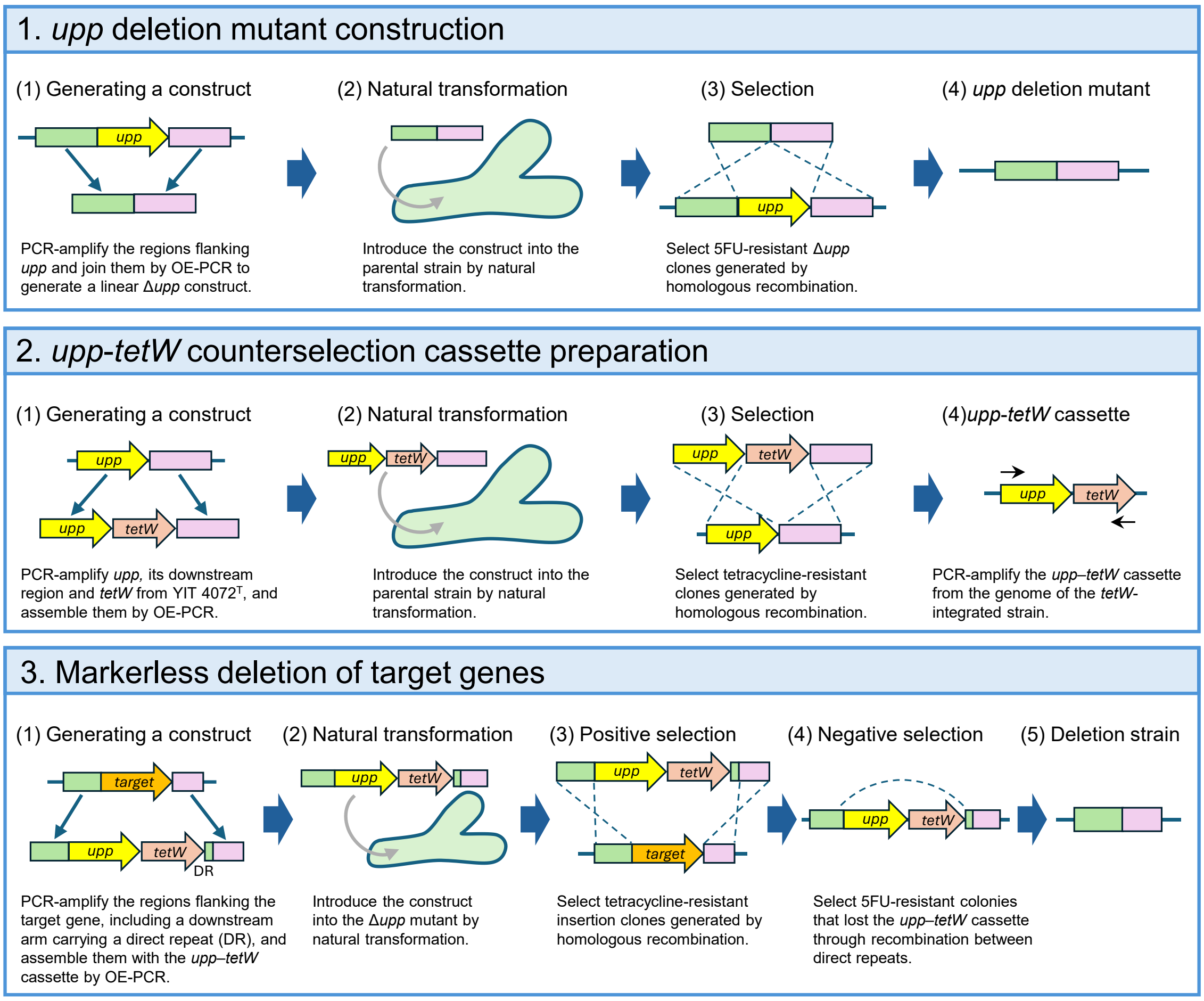

OE-PCR: overlap-extension PCR, DR: direct repeat, 5FU: 5-fluorouracil.

Figure S7. Overall workflow of markerless genome manipulation

Alt text: Three-step workflow for markerless gene deletion using *upp* counterselection through homologous recombination and a transient *tetW* selection cassette.

Supplementary tables

Table S1. Donor–recipient strain combinations and selective conditions used to generate recombinant strains

Pairwise SNPs, aligned length, and SNPs/kb indicate the sequence divergence between each donor–recipient pair.

Table S2. Natural transformation across *Bifidobacterium pseudocatenulatum* isolates

Twenty-seven *B. pseudocatenulatum* isolates from our strain collection were tested for the acquisition of a *tetW*-carrying purified DNA fragment. The occurrence of tetracycline-resistant colonies following exposure to the purified DNA is indicated as positive (+) or negative (−).

Table S3. Genes upregulated under conditions with a high natural transformation frequency in *Bifidobacterium pseudocatenulatum* YIT 12819

Genes displaying more than a 10-fold expression increase under high natural transformation frequency-associated conditions (50 mM $MgCl_2$) compared with those associated with a low natural transformation frequency (pH 6) in strain YIT 12819.

Table S4. Phenotypic characteristics of strains included in the strain library

LCX utilisation phenotypes were assigned based on previously reported data [9]. Starch utilisation was assessed by increased turbidity indicative of growth in medium containing starch as the sole carbohydrate source. Resistance to tetracycline and erythromycin was evaluated by colony formation on tetracycline- and erythromycin-supplemented agar (5 and 1 μg/mL, respectively). (+): positive growth or resistance. (−): no detectable growth or sensitive under the tested conditions.

Table S5. Long-arm donor construction primers

Table S6. *upp* deletion mutant construction primers

Table S7. *upp–tetW* counterselection cassette construction primers

Table S8. Natural-transformation-defective deletion mutant construction primers
